# A comparative study for optimizing photocatalytic activity of TiO_2_-based composites with ZrO_2_, ZnO, Ta_2_O_5_, SnO, Fe_2_O_3_, and CuO additives

**DOI:** 10.1038/s41598-024-77752-5

**Published:** 2024-11-08

**Authors:** I. Abdelfattah, A. M. El-Shamy

**Affiliations:** 1 Water Pollution Research Department, National Research Centre, El-Bohouth St. 33, Dokki, P.O. 12622, Giza, Egypt; 2 Physical Chemistry Department, Electrochemistry and Corrosion Lab, National Research Centre, El-Bohouth St. 33, Dokki, P.O. 12622, Giza, Egypt

**Keywords:** Photocatalysis, Metal oxide composites, Imazapyr, Environmental remediation, Charge separation, Energy conservation, Environmental sciences, Environmental social sciences, Natural hazards

## Abstract

Despite the widespread use of titanium dioxide (TiO_2_) in photocatalytic applications, its inherent limitations, such as low efficiency under visible light and rapid recombination of electron-hole pairs, hinder its effectiveness in environmental remediation. This study presents a comparative investigation of TiO_2_-based composites, including TiO_2_/ZrO_2_, ZnO, Ta_2_O_3_, SnO, Fe_2_O_3_, and CuO, aiming to assess their potential for enhancing photocatalytic applications. Photocatalysis holds promise in environmental remediation, water purification, and energy conversion, with TiO_2_ being a prominent photocatalyst. To improve efficiency and broaden applicability, various metal oxide composites have been explored. Composites were synthesized and characterized using techniques such as XRD, SEM, TEM, and zeta potential analysis to evaluate their structural and morphological properties. Photocatalytic performance was assessed by degrading herbicide Imazapyr under UV illumination. Results revealed that, the photo-activity of all prepared composites were more effective than the photo-activity of commercial hombikat UV-100. The photonic-efficiency is arranged according to the order TiO_2_/CuO > TiO_2_/SnO > TiO_2_/ZnO > TiO_2_/Ta_2_O_3_ > TiO_2_/ZrO_2_ > TiO_2_/Fe_2_O_3_ > Hombikat TiO_2_-UV100. All composites exhibited superior performance, attributed to enhanced light absorption and charge separation. The study underscores the potential of these composites for environmental remediation and energy conservation, offering valuable insights for the development of advanced photocatalysts.

## Introduction

The field of photocatalysis has garnered significant attention in recent years due to its promising applications in environmental remediation, water purification, and energy conversion^1^. Photocatalysts, in the best utilizations, are materials that can harness solar energy to catalyze chemical reactions, making them a sustainable and eco-friendly option for addressing various global challenges. Among photocatalysts, titanium dioxide (TiO_2_) stands out as one of the most extensively studied and widely used materials due to its excellent photocatalytic properties^2^. TiO_2_ as a photocatalyst, with its wide bandgap and strong oxidative properties, is a well-known and effective photocatalyst. When exposed to ultraviolet (UV) light, TiO_2_ generates electron-hole pairs that can participate in redox reactions, leading to the degradation of organic pollutants and the production of reactive oxygen species (ROS)^3^. These ROS are highly effective in breaking down a wide range of organic compounds, making TiO_2_ an attractive option for wastewater treatment and air purification^4^. While TiO_2_ is a powerful photocatalyst, its efficiency is limited by factors such as rapid electron-hole recombination and a relatively large bandgap, restricting its activity to UV light^5^. To overcome these limitations, researchers have explored various strategies, including doping with other elements, and developing composite materials. Composite photocatalysts involve combining TiO_2_ with other materials, such as metal oxides, to create synergistic effects that enhance photocatalytic performance^6^. These composites offer the advantage of improved light absorption, charge separation, and increased surface area, all of which contribute to enhance photocatalysis^7^. In the context of this study, TiO_2_ was combined with several metal oxides, including ZrO_2_, ZnO, Ta_2_O_3_, SnO, Fe_2_O_3_, and CuO, to investigate their potential for boosting photocatalytic applications. It can serve as a co-catalyst, promoting charge separation and extending photocatalysis into the visible light range^8^. This synergy between TiO_2_ and these metal oxides holds promise for improving the overall efficiency of photocatalytic reactions. can enhance charge separation, and inhibit electron-hole recombination^9,10^. This co-catalyst has the potential to improve the efficiency of photocatalytic reactions. Characterization techniques, to assess the structural, morphological, and surface properties of these TiO_2_-based composite photocatalysts, a range of characterization techniques, including X-ray diffraction (XRD), scanning electron microscopy (SEM), energy-dispersive X-ray spectroscopy (EDS), transmission electron microscopy (TEM), and zeta potential analysis, were employed^11–14^. These techniques provide valuable insights into the composition and behavior of composites. Photocatalytic evaluation, the photocatalytic activities of these composite materials were evaluated by studying the degradation of the sample of organic pollutants under both UV and visible light irradiation^15^. The global population continues to grow rapidly, leading to increased food demand and placing significant pressure on food production systems. To protect crops from the detrimental effects of weeds and grasses, farmers widely utilize herbicides^16^. However, the extensive use of these chemicals has resulted in their accumulation in the environment, presenting a serious concern due to their toxic and harmful effects on both ecosystems and human health. This alarming reality has prompted researchers to seek alternatives to these hazardous substances to mitigate their adverse impacts^17^. Imazapyr, scientifically known as [(RS)-2-(4-methyl-5-oxo-4-propan-2-yl-1 H-imidazol-2-yl) pyridine-3-carboxylic acid], is a nonselective herbicide primarily used for controlling grasses. While it is not intended for aquatic applications, it has been detected on water surfaces due to spray drift, and it can penetrate deep into groundwater, posing significant environmental risks^18^. In soil, Imazapyr can persist for 6 to 12 months without breaking down, and its high solubility in water means that even minimal soil concentrations around 0.05 mg/kg can be harmful to plants. The European Union has set a maximum allowable limit for herbicides and pesticides in drinking water at just 0.1 µg/L, highlighting the potential dangers associated with Imazapyr’s presence in the environment^19^. The removal of imazapyr has been explored through various methods, including electrochemical oxidation, adsorption, and chemical oxidation. However, traditional chemical oxidation methods such as using chlorine dioxide, ozone, and chlorine have notable drawbacks; specifically, the secondary byproducts generated during these processes can be toxic^20^. In contrast, Advanced Oxidation Processes (AOPs) represent a promising new approach that relies on the generation of hydroxyl (^•^OH) radicals to effectively degrade organic compounds. Among these techniques, heterogeneous photocatalysis using titanium dioxide (TiO_2_) has emerged as one of the most efficient methods, offering high productivity while minimizing energy loss in treatment applications^21^. Recent studies have extensively evaluated the photocatalytic degradation of imazapyr using commercial TiO_2_ and TiO_2_ modified with mesoporous structures, as well as with additives like aluminum oxide (Al_2_O_3_) and tungsten oxide (WO_3_)^22^. However, to our knowledge, there are currently no reports examining the photodegradation of imazapyr using mesoporous gallium oxide-titanium dioxide (Ga_2_O_3_-TiO_2_) nanocomposites^23^. Therefore, conducting a fundamental study to investigate the relationship between the composition of Ga_2_O_3_ in Ga_2_O_3_-TiO_2_ nanocomposites and their effectiveness in photodegradation is crucial and warrants further exploration^24^. Our research focuses on optimizing the material’s composition and structure to maximize its efficiency in harnessing solar energy for catalytic reactions, thus facilitating the degradation of pollutants or the production of valuable chemicals. What sets our research apart from others in the field is the innovative approach we have taken^25^. Firstly, our synthesis method is designed to be cost-effective, utilizing readily available and affordable starting materials without compromising on the quality or performance of the final product. This ensures scalability and accessibility, making our photocatalyst suitable for widespread practical applications^26^. Moreover, the novelty of our work lies in the novel architecture and composition of the synthesized material, which endows it with superior photocatalytic activity compared to existing alternatives. Our research explores new avenues for enhancing photocatalyst performance, such as surface engineering, doping strategies, or heterostructure design, to achieve unprecedented levels of efficiency and selectivity in catalytic processes^27^. The primary motivation behind our research is to bridge a critical gap in the existing knowledge concerning TiO_2_-based photocatalysts and their potential for environmental remediation and energy conservation^28^. In summary, our research fills a significant gap in the literature by providing a comparative analysis of TiO_2_-based composites and identifying promising candidates for advancing photocatalytic applications. We believe that our study contributes valuable insights to the field and sets the stage for further research in the development of advanced photocatalysts^29^. In addition, our research lies in the systematic comparison of TiO_2_-based composites with six different metal oxide additives ZrO_2_, ZnO, Ta_2_O_5_, SnO, Fe_2_O_3_, and CuO under identical experimental conditions^30–35^. Unlike previous studies that typically focus on one or two additives or different experimental parameters, our work offers a comprehensive analysis, providing insights into how each additive affects the photocatalytic performance of TiO_2_. Because of its significant oxidative potential, stability, and lack of toxicity, titanium dioxide (TiO_2_) is one of the materials for photocatalysis that has been explored the most. However, its practical use is limited by a number of fundamental problems, especially when it comes to using solar energy. The main issues with TiO_2_-based photocatalysts are covered below, along with potential solutions to increase photocatalytic efficiency. TiO_2_ is only active in the ultraviolet (UV) portion of the solar spectrum due to its wide bandgap, which is roughly 3.0 eV for rutile and 3.2 eV for anatase. Only around 5% of solar energy is converted into UV light, meaning that most visible light is wasted. Dopants like nitrogen (N), carbon (C), or sulphur (S) could be added to the first potential solutions in order to reduce the bandgap. The high rate of electron-hole recombination, which drastically lowers the overall efficiency, is one of the main issues with TiO_2_-based photocatalysis. The lifespan of charge separation is limited because the photogenerated electrons and holes frequently recombine before taking part in photocatalytic events. By embedding noble metals like gold (Au) or silver (Ag) onto TiO_2_, the initial potential solution is produced. This accelerates the creation of hot electrons and improves charge separation by inducing SPR. By producing localized electric fields, these metal nanoparticles lessen electron-hole recombination. Building heterojunctions between TiO_2_ and other semiconductors, such as ZnO, g-C_3_N₄, or SnO_2_, is the basis of the second solution, which aims to enhance charge separation. Recombination is reduced in these structures because the photogenerated electrons and holes are spatially separated. In order to complete the third, cocatalysts must be loaded. Since the number of active sites available for photocatalytic processes is directly proportional to the surface area, the surface area of TiO_2_ also limits its photocatalytic performance. The efficiency of bulk TiO_2_ structures is often diminished by their comparatively small surface area. The manufacture of TiO_2_ nanoparticles, nanorods, or nanotubes yields the first potential solution by greatly increasing the surface area and exposing more active sites for photocatalytic processes. Additionally, improved light absorption and charge transfer are made possible by nano structuring. The second is relying on the use of mesoporous TiO_2_ structures, which offer a high surface area with well-organized pores, improving light-harvesting capacity and reactant accessibility to active areas. The surface reaction kinetics of TiO_2_ are frequently slow, especially for water oxidation or reduction reactions, even with effective charge separation. The total photocatalytic activity is limited by this slow reaction rate. By offering active sites for catalytic activities, cocatalysts such as Pt, RuO_2_, or Co-Pi can speed up surface reactions on the surface of TiO_2_. This is the first potential solution. These cocatalysts improve oxidation (like O_2_ evolution) and reduction (like H_2_ synthesis) reactions. The second one focusses on altering the surface of TiO_2_ with metal complexes or organic compounds to enhance reactant adsorption and promote quicker surface reactions. Additionally, this functionalization can improve selectivity for processes. TiO_2_-based photocatalysts have a lot of potential for energy and environmental applications, but they also have a lot of drawbacks, including sluggish surface reaction kinetics, a large bandgap, significant electron-hole recombination, and a small surface area. These restrictions can be overcome, although, by employing sophisticated techniques such doping, composite synthesis, heterojunction engineering, cocatalyst loading, and nano structuring. TiO_2_-based photocatalysts can be greatly enhanced by using these solutions, opening the door to more effective and useful uses in environmental remediation, water treatment, and solar energy utilization. This study not only explores the synergetic effects of the additives but also delves deeper into the mechanistic understanding of their impact on photocatalytic efficiency, surface interactions, and stability. Furthermore, our approach uniquely combines both structural and performance evaluations to optimize the photocatalytic process^36^. Compared to previously cited work, our research stands out by offering a broader comparative scope, more detailed mechanistic insights, and identifying specific composites with tailored properties for enhanced photocatalytic applications. This positions our work as a valuable contribution to advancing the field of photocatalysis^37^. The common goals of sustainability, less environmental impact, and resource efficiency make the relationship between environmental remediation and energy applications clear. The development of sophisticated materials and techniques, energy recovery from trash, and the incorporation of renewable energy into remediation technologies are fostering a more cooperative approach to tackling the twin problems of energy demand and environmental deterioration. As more research is done, advancements in this field should offer strong answers for a more sustainable future in which trash is not only cleaned but also converted into a useful energy source, lessening the ecological impact of humanity as a whole.

## Materials and methods

### Materials

Tetrabutyl orthotitanate Ti[OC(CH_3_)_3_]_4_(TBOT) Sigma-Aldrich, Zirconium (IV) acetylacetonate (C_20_H_28_O_8_Zr) Merck, hydrated zinc nitrate (Zn(NO_3_)_2_·6H_2_O) Sigma-Aldrich, Tantalum pentachloride (TaCl_5_) Sigma-Aldrich, Tin chloride (SnCl_4_·5H_2_O), Ferric acetylacetonate (Fe(C_5_H_7_O_2_)_3_) Sigma-Aldrich, Copper(II) nitrate hemi (pentahydrate) (Cu(NO_3_)_2_·2.5H_2_O), Imazapyr (C_13_H_15_N_3_O_3_ > 99%), HCl, C_2_H_5_OH, CH_3_OH, H_3_PO_4_, and CH_3_COOH, the triblock copolymer surfactant EO106-PO70EO106(F-127), MW12600 g/mol), were purchased from Sigma-Aldrich. Commercial TiO_2_ (Hombikat UV-100, 100% anatase and 230 m^2^g^− 1^ surface area) was kindly provided by Sachtleben Chemie GmbH.

### Preparation of mesoporous ZrO2-TiO2, ZnO-TiO2, Ta2O3-TiO2, SnO2-TiO2, Fe2O3-TiO2 and CuO-TiO2, nanocomposites

Different mesoporous nanocomposites were prepared via sol-gel process using F127 triblock copolymer. Typically, 1.6 g of F127 was added to 30 mL of ethanol while stirring for 60 min, and then 0.74 mL of HCl, 3.5 mL of TBOT and 2.3 mL of CH_3_COOH were added drop wise to F127 solution under magnetic stirring for 30 min. The calculated amount of Zirconium (IV) acetylacetonate (C_20_H_28_O_8_Zr) and other precursors was added to the mixture mesophase (F127-TBOT- CH_3_COOH) vigorously stirring for 60 min to obtain 0.5, 1, 2, 3 and 5 wt% ZrO_2_-TiO_2_ nanocomposites. The prepared mesophase was put into 40% humidity chamber at 40°C for 12 h to evaporate C_2_H_5_OH and form gel and dried at 65 °C for 24 h. Afterward, it was calcined at 500 °C in air at a heating rate of 1°C/min to reach 500 °C for 4 h to remove the template and to different produce mesoporous nanocomposites at different oxides content^38–41^.

### Photocatalytic activity tests

0.05 g photocatalyst and 10 mM KNO_3_ was added in 50 mL of water and then it was sonicated in an ultrasonic cleaning bath for 15 min to disperse the photocatalyst. KNO_3_ was added to keep the ionic strength of the solution to equivalent the excess of HCl (pH = 4). The imazapyr concentration [0.08 mmol L^− 1^] was maintained to carry out the experimental work^42^. To reach adsorption equilibrium, the imazapyr and photocatalyst were kept under continuous magnetically stirring at 300 rpm for 4 h at 25 ± 1°C. Illumination experiments were conducted under top irradiation of a borosilicate glass beaker and the photonic flow was ρ = 2 m Wcm^− 2^. The samples were taken at regular time intervals for analysis. The analysis of imazapyr concentrations was measured through high performance liquid chromatography (HPLC) system from Agilent Technologies 1260 Infinity composed of a G1311C-1260 Quat pump and a G1365D-1260 MWD UV Detector adjusted to 254 nm. An Agilent Eclipse plus C18 column employing at room temperature was performed as stationary phase, and a mixture of water and methanol (70:30%v/v) employing as mobile phase at pH value ∼3 by adding H_3_PO_4_. The flow rate was fixed at 0.8 mLmin^− 1^ and the retention time at 4.60 min^43^. A calibration curve (R^2^ = 0.9996) was determined from the patrons of 6 different concentration analysis at range 0–0.08 mmol L^− 1^. The HPLC analysis measured 2–3 replicates, allowing initial reaction rates to be considered with a mean experimental error ± 5%. This error is determined to be the sum of the HPLC instrument error and the error intrinsic to mathematical calculations from the experimental concentration as a function of time plots. The initial rate for imazapyr photodegradation was calculated through the first 30 min of UV irradiation. The photonic efficiency was calculated as given in the following Eqs^44–47^.

$$\upvarepsilon = {\text{r}}*100/{\text{I}}$$

where ɛ is the photonic efficiency (%), r the photodegradation rate of imazapyr (mol L^− 1^s^− 1^), and I the incident photon flux (7.03 × 10^− 6^ Ein L^− 1^s^− 1^). The UV-A incident photon flow was measured by ferrioxalate actinometry.

### Characterization of composite photocatalysts

#### **Structural analysis of TiO**_**2**_**-based composites**

Structural analysis plays a crucial role in understanding the composition, morphology, crystallinity, and phase distribution within TiO_2_-based composites. Various analytical techniques are employed to characterize these properties in detail. X-ray Diffraction (XRD) is used to determine the crystallographic structure and phase composition of the composite material. The sample is exposed to X-rays, and the resulting diffraction pattern is recorded. By analyzing the diffraction peaks, the crystalline phases present, their crystal structure, and lattice parameters can be identified. Phase identification, crystallite size, lattice parameters, and degree of crystallinity^48^. Scanning Electron Microscopy (SEM) provides high-resolution images of the surface morphology and microstructure of the composite. A focused electron beam scans the sample surface, and the interaction of electrons with the sample generates signals that are used to create images. Surface morphology, particle size, shape, distribution, and agglomeration of nanoparticles^49^. Transmission Electron Microscopy (TEM) provides detailed information about the internal structure, size, and shape of nanoparticles at the atomic level. A focused electron beam is transmitted through an ultrathin sample, and the resulting electron diffraction and imaging provide high-resolution images of the sample’s internal structure. Nanoparticle size, morphology, crystallinity, lattice structure, and interfacial interactions. Zeta potential analysis is used to determine the surface charge and stability of colloidal suspensions. The electrophoretic mobility of charged particles in a suspension is measured, and the zeta potential is calculated, providing information about particle stability and interactions. Surface charge, colloidal stability, and interactions between nanoparticles in the composite^50^. These structural analysis techniques provide comprehensive insights into the composition, morphology, crystallinity, and surface properties of TiO_2_-based composites, enabling researchers to optimize their synthesis and tailor their properties for specific applications in photocatalysis, environmental remediation, and energy conversion^51^.

#### Data analysis

The results obtained from the photocatalytic experiments were statistically analyzed to evaluate the photocatalytic performance of the composite materials. Comparative analyses were conducted to determine the synergistic effects of the metal oxide composites. his comprehensive methodology enabled the synthesis, characterization, and evaluation of TiO_2_-based composites with ZrO_2_, ZnO, Ta_2_O_3_, SnO, Fe_2_O_3_, and CuO for enhanced photocatalytic applications, providing valuable insights into their potential for environmental remediation and energy conversion^52^.

## Results and discussion

### Change in imazapyr concentration as a function of illumination time

#### Mesoporous CuO-TiO2 nanocomposites

**Fig. 1:**
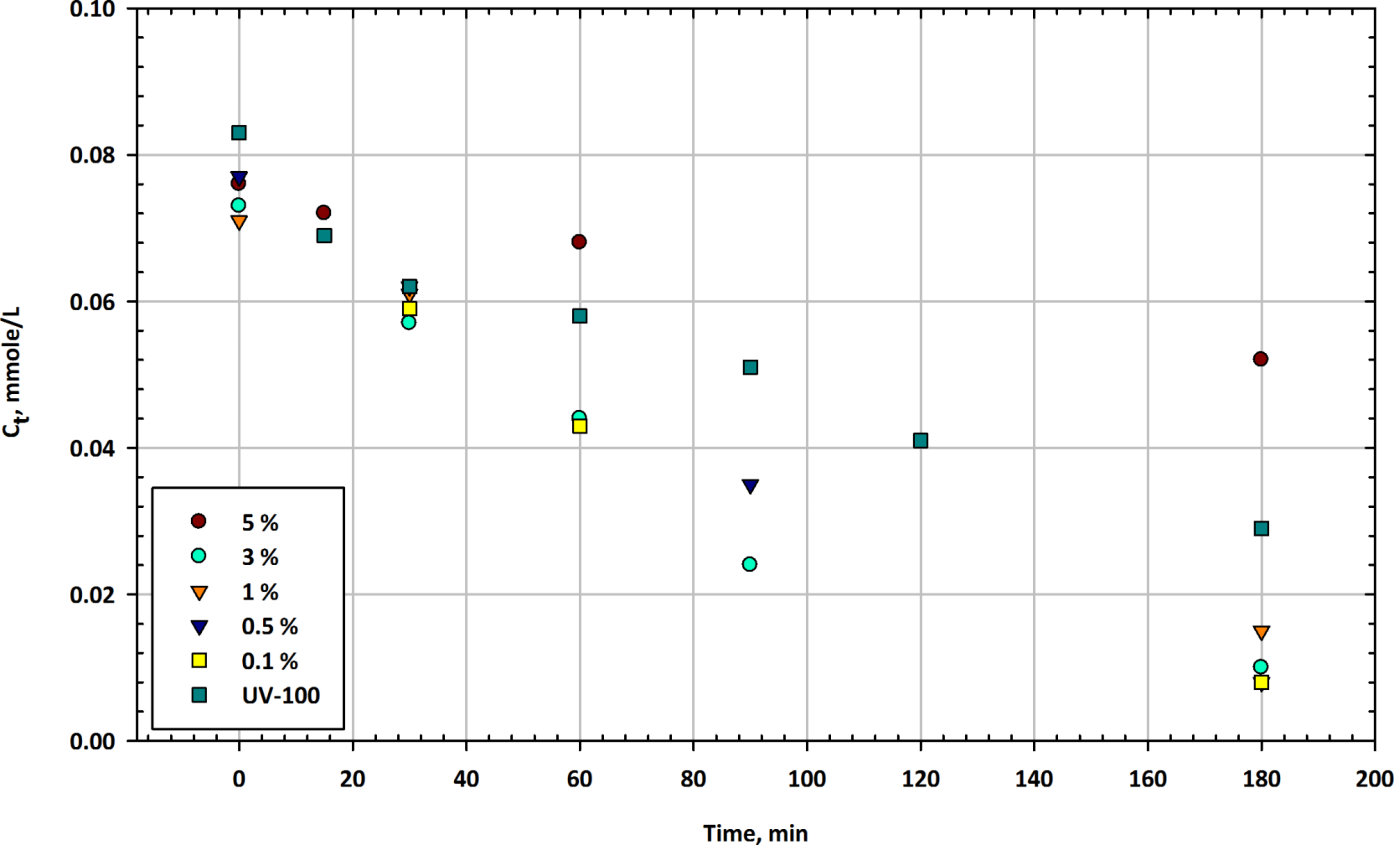
Change in imazapyr concentration as a function of illumination time in the presence of mesoporous CuO-TiO_2_ nanocomposites at different CuO contents compared with Hombikat UV-100 as a reference.

Figure 1 presents the changes in imazapyr concentration (C_t_) as a function of illumination time in the presence of mesoporous CuO-TiO_2_ nanocomposites at different CuO contents, along with Hombikat UV-100 as a reference catalyst. The t Fig. 1 shows the residual concentration of the herbicide at various time intervals. Initial Concentration (0 min), the initial concentration of imazapyr in the solution is approximately 0.076 mmol/L when no catalyst is present. In the presence of CuO-TiO_2_ nanocomposites, the initial concentrations vary with different CuO contents, ranging from 0.071 mmol/L to 0.083 mmol/L^53^. The reference catalyst, UV-100, shows an initial concentration of 0.083 mmol/L. After 15 min of illumination, the imazapyr concentration decreases to 0.072 mmol/L for the reference UV-100 catalyst. Among the CuO-TiO_2_ nanocomposites, only the 0.1% CuO content shows a decrease, reaching 0.069 mmol/L. At the 30-minute mark, the reference UV-100 catalyst continues to reduce imazapyr concentration to 0.057 mmol/L. Among the CuO-TiO_2_ nanocomposites, the 0.5% CuO content now shows a decrease to 0.062 mmol/L, while the 3% and 5% CuO contents are at 0.061 mmol/L and 0.062 mmol/L, respectively. After 60 min, the reference catalyst UV-100 achieves a concentration of 0.043 mmol/L, showing a significant reduction. The 0.5% CuO content among the nanocomposites also reduces imazapyr concentration to 0.043 mmol/L. At 90 min, the reference catalyst UV-100 reaches a concentration of 0.051 mmol/L^54^. Among the nanocomposites, only the 3% CuO content reduces the concentration to 0.035 mmol/L. After 120 min, the reference UV-100 catalyst further reduces the concentration to 0.041 mmol/L. At the end of the 180-minute test, the reference catalyst UV-100 achieves a concentration of 0.029 mmol/L. Among the CuO-TiO_2_ nanocomposites, the 0.1% CuO content also reduces the concentration to 0.029 mmol/L. The data in Fig. 1 demonstrate the influence of both the CuO content in the nanocomposites and the illumination time on the degradation of imazapyr. The reference catalyst UV-100 consistently shows a reduction in imazapyr concentration over time, reaching the lowest concentration after 180 min. Among the nanocomposites, the 0.5% CuO content appears to be the most effective in degrading imazapyr, achieving similar concentrations as UV-100 at various time intervals^55^. Interestingly, the performance of the nanocomposites is not strictly linear with increasing CuO content, as different CuO contents show variations in activity. These results suggest that the CuO-TiO_2_ nanocomposites have the potential to be effective catalysts for the degradation of imazapyr, and further optimization may be required to determine the ideal CuO content for maximum efficiency. Additionally, the data highlights the importance of illumination time, with longer exposure times leading to lower imazapyr concentrations, indicating the potential for the photocatalytic degradation of this herbicide^56^.

Table 1 presents the time-dependent residual concentrations (C_t_ in mmol/L) of herbicide for different catalyst concentrations (5%, 3%, 1%, 0.5%, 0.1%) and UV100 treatment. The values reflect the mean concentration ± standard error. Gaps in the data indicate measurements that were not taken at those time intervals. The results demonstrate the degradation profile of the herbicide under varying catalyst conditions.

**Table 1:** Residual concentration of herbicide over time for different catalyst percentages with UV irradiation (UV100) and corresponding error margins.

Min	5%	3%	1%	0.5%	0.1%	UV100
0	0.076 ± 0.003	0.073 ± 0.003	0.071 ± 0.003	0.077 ± 0.003	0.083 ± 0.004	0.083 ± 0.004
15	0.072 ± 0.003	–	–	–	0.069 ± 0.003	–
30	–	0.057 ± 0.003	0.061 ± 0.003	0.062 ± 0.003	0.059 ± 0.003	0.062 ± 0.003
60	0.068 ± 0.003	0.044 ± 0.003	–	–	0.043 ± 0.002	0.058 ± 0.003
90	–	0.024 ± 0.002	–	0.035 ± 0.002	–	0.051 ± 0.003
120	–	–	–	–	0.041 ± 0.002	–
180	0.052 ± 0.003	0.01 ± 0.002	0.015 ± 0.002	0.008 ± 0.002	0.008 ± 0.002	0.029 ± 0.002

#### Mesoporous Fe2O3-TiO2 nanocomposites

**Fig. 2:**
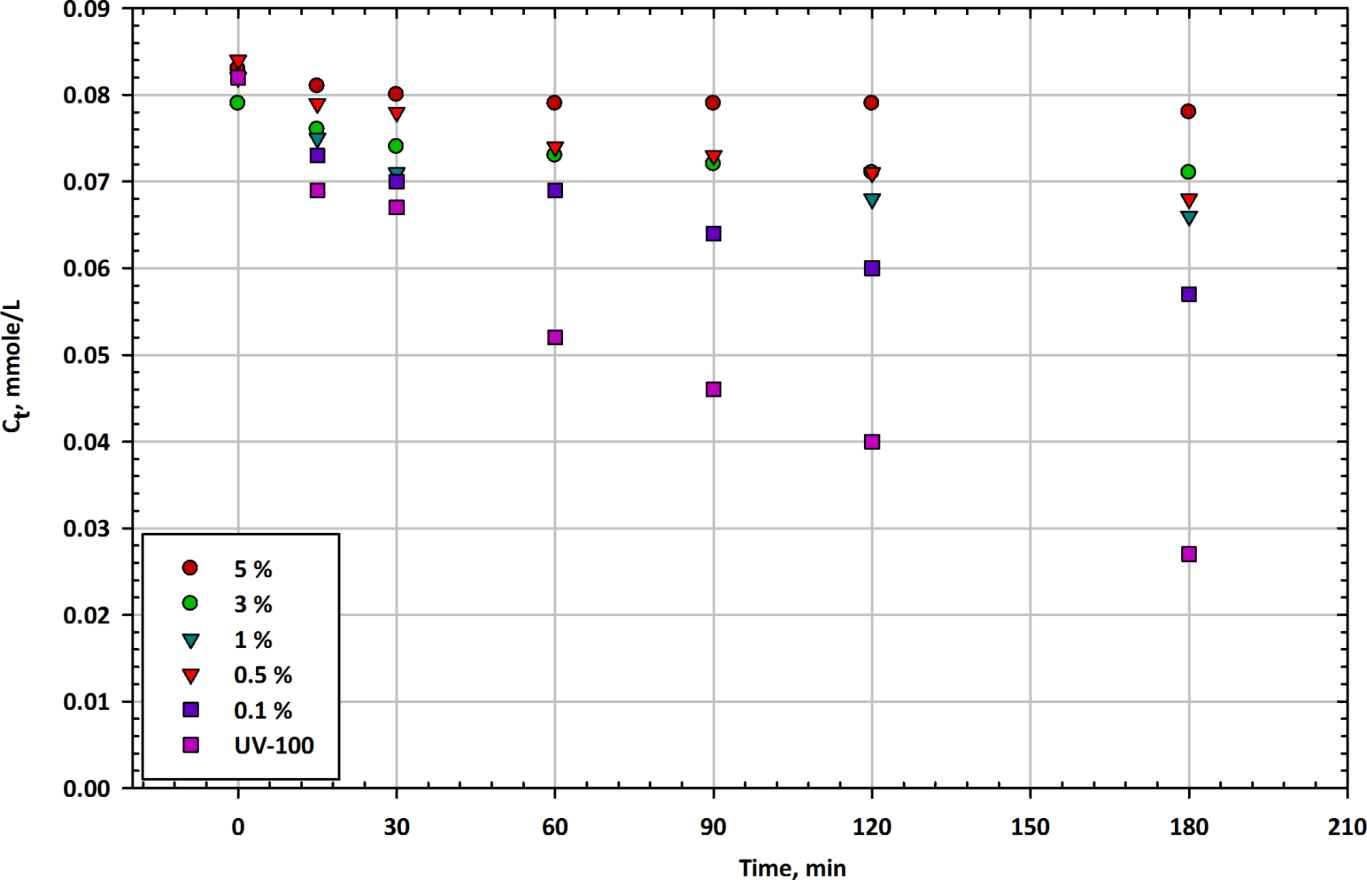
Change in imazapyr concentration as a function of illumination time in the presence of mesoporous Fe_2_O_3_-TiO_2_ nanocomposites at different Fe_2_O_3_ contents compared with Hombikat UV-100 as a reference.

Figure 2 presents the changes in imazapyr concentration (Ct) as a function of illumination time in the presence of mesoporous Fe_2_O_3_-TiO_2_ nanocomposites at different Fe_2_O_3_ contents, along with Hombikat UV-100 as a reference catalyst. The table displays the residual concentration of the herbicide at various time intervals^57^. At the start of the experiment, the initial concentration of imazapyr in the solution is approximately 0.083 mmol/L when no catalyst is present. In the presence of Fe_2_O_3_-TiO_2_ nanocomposites, the initial concentrations vary with different Fe_2_O_3_ contents, ranging from 0.075 mmol/L to 0.084 mmol/L. The reference catalyst, UV-100, shows an initial concentration of 0.082 mmol/L. After 15 min of illumination, all Fe_2_O_3_-TiO_2_ nanocomposites show a reduction in imazapyr concentration, with the 0.5% Fe_2_O_3_ content exhibiting the lowest concentration at 0.073 mmol/L. The reference UV-100 catalyst also reduces the concentration to 0.069 mmol/L. At the 30-minute mark, all Fe_2_O_3_-TiO_2_ nanocomposites continue to reduce imazapyr concentration, with the 0.5% Fe_2_O_3_ content having the lowest concentration at 0.067 mmol/L. The reference UV-100 catalyst achieves a concentration of 0.071 mmol/L^58^. After 60 min, imazapyr concentrations continue to decrease for all catalysts, with the 0.5% Fe_2_O_3_ content exhibiting the lowest concentration at 0.052 mmol/L. The reference UV-100 catalyst achieves a concentration of 0.074 mmol/L. At 90 min, imazapyr concentrations further decreased for all catalysts, with the 0.5% Fe_2_O_3_ content having the lowest concentration at 0.046 mmol/L. The reference UV-100 catalyst achieves a concentration of 0.073 mmol/L. After 120 min, imazapyr concentrations continue to decrease, with the 0.5% Fe_2_O_3_ content exhibiting the lowest concentration at 0.040 mmol/L. The reference UV-100 catalyst achieves a concentration of 0.071 mmol/L. At the end of the 180-minute test, imazapyr concentrations continue to decrease for all catalysts, with the 0.5% Fe_2_O_3_ content having the lowest concentration at 0.027 mmol/L. The reference UV-100 catalyst achieves a concentration of 0.057 mmol/L^59^. Figure 2 demonstrates the impact of different Fe_2_O_3_ contents in mesoporous Fe_2_O_3_-TiO_2_ nanocomposites on the degradation of imazapyr over time. Generally, all Fe_2_O_3_-TiO_2_ nanocomposites show the ability to reduce imazapyr concentrations, with the 0.5% Fe_2_O_3_ content consistently achieving the lowest concentrations at various time intervals. The reference catalyst, UV-100, also exhibits degradation capabilities but tends to have slightly higher imazapyr concentrations compared to the 0.5% Fe_2_O_3_ nanocomposite^60^. These findings suggest that Fe_2_O_3_-TiO_2_ nanocomposites have photocatalytic activity in degrading imazapyr, and the presence of Fe_2_O_3_ content influences their efficiency. The results also indicate that longer illumination times lead to lower imazapyr concentrations, supporting the potential for photocatalytic degradation of this herbicide. Further research may be needed to optimize the Fe_2_O_3_ content and other experimental parameters to enhance the photocatalytic activity of these nanocomposites for herbicide removal applications^61^.

Table 2 illustrates the time-dependent degradation of herbicide residual concentration (Ct in mmol/L) across different catalyst percentages (100%, 5%, 3%, 1%, 0.5%, 0.1%) and UV100 exposure. The results are presented as mean concentrations with associated error margins, reflecting variability in the data. This comparison highlights the effectiveness of different catalyst levels and UV treatment in reducing herbicide concentration over time.

**Table 2:** Herbicide residual concentration (C_t_) over time with various catalyst concentrations and UV100 treatment: Mean Values ± Error Margins.

Min	100%	5%	3%	1%	0.5%	0.1%	UV100
0	0.083 ± 0.003	0.079 ± 0.003	0.082 ± 0.003	0.084 ± 0.003	0.082 ± 0.003	0.082 ± 0.003	0.083 ± 0.003
15	0.081 ± 0.003	0.076 ± 0.003	0.075 ± 0.003	0.079 ± 0.003	0.073 ± 0.003	0.069 ± 0.003	0.069 ± 0.003
30	0.8 ± 0.003	0.074 ± 0.003	0.071 ± 0.003	0.078 ± 0.003	0.07 ± 0.003	0.067 ± 0.003	0.062 ± 0.003
60	0.079 ± 0.003	0.073 ± 0.003	0.074 ± 0.003	0.074 ± 0.003	0.069 ± 0.003	0.052 ± 0.002	0.058 ± 0.003
90	0.079 ± 0.003	0.072 ± 0.003	0.073 ± 0.003	0.073 ± 0.003	0.064 ± 0.003	0.046 ± 0.002	0.051 ± 0.003
120	0.079 ± 0.003	0.071 ± 0.003	0.068 ± 0.003	0.071 ± 0.003	0.06 ± 0.003	0.04 ± 0.002	0.041 ± 0.002
180	0.078 ± 0.003	0.071 ± 0.003	0.066 ± 0.003	0.068 ± 0.003	0.057 ± 0.003	0.027 ± 0.002	0.029 ± 0.002

#### Mesoporous SnO-TiO2 nanocomposites

**Fig. 3:**
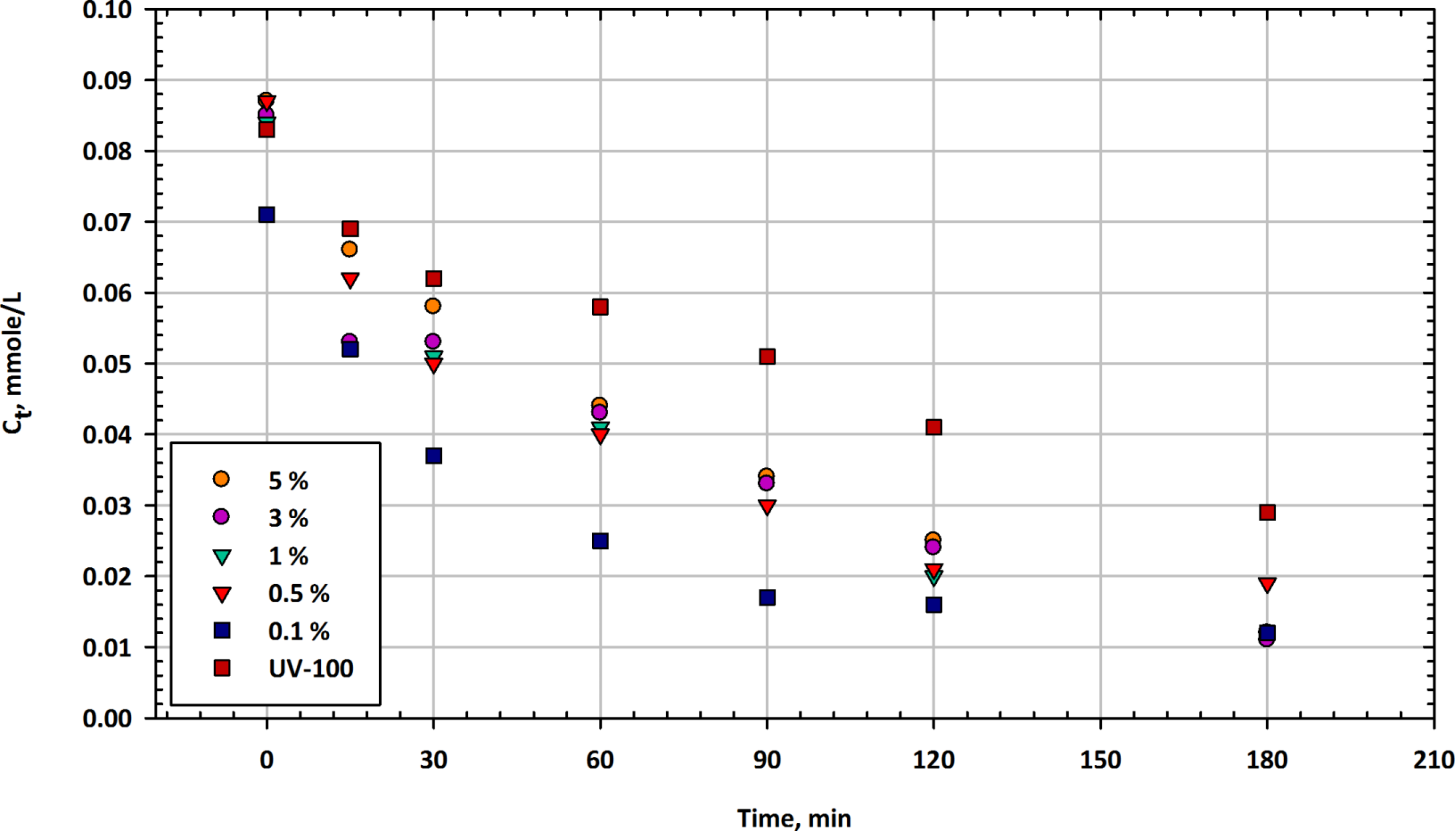
Change in imazapyr concentration as a function of illumination time in the presence of mesoporous SnO-TiO_2_ nanocomposites at different SnO contents compared with Hombikat UV-100 as a reference.

Figure 3 presents the changes in imazapyr concentration (C_t_) as a function of illumination time in the presence of mesoporous SnO-TiO_2_ nanocomposites at different SnO contents, along with Hombikat UV-100 as a reference catalyst. The table displays the residual concentration of the herbicide at various time intervals. At the beginning of the experiment, the initial concentration of imazapyr in the solution is approximately 0.087 mmol/L when no catalyst is present. In the presence of SnO-TiO_2_ nanocomposites, the initial concentrations vary with different SnO contents, ranging from 0.071 mmol/L to 0.085 mmol/L^62^. The reference catalyst, UV-100, shows an initial concentration of 0.083 mmol/L. After 15 min of illumination, all SnO-TiO_2_ nanocomposites show a significant reduction in imazapyr concentration, with the 0.1% SnO content achieving the lowest concentration at 0.052 mmol/L. The reference UV-100 catalyst also reduces the concentration to 0.069 mmol/L. At the 30-minute mark, all SnO-TiO_2_ nanocomposites continue to reduce imazapyr concentration, with the 0.1% SnO content having the lowest concentration at 0.037 mmol/L. The reference UV-100 catalyst achieves a concentration of 0.062 mmol/L. After 60 min, imazapyr concentrations continue to decrease for all catalysts, with the 0.1% SnO content exhibiting the lowest concentration at 0.025 mmol/L. The reference UV-100 catalyst achieves a concentration of 0.058 mmol/L^63^. At 90 min, imazapyr concentrations further decreased for all catalysts, with the 0.1% SnO content having the lowest concentration at 0.017 mmol/L.

The reference UV-100 catalyst achieves a concentration of 0.051 mmol/L. After 120 min, imazapyr concentrations continue to decrease, with the 0.1% SnO content exhibiting the lowest concentration at 0.016 mmol/L. The reference UV-100 catalyst achieves a concentration of 0.041 mmol/L. At the end of the 180-minute test, imazapyr concentrations continue to decrease for all catalysts, with the 0.1% SnO content having the lowest concentration at 0.012 mmol/L. The reference UV-100 catalyst achieves a concentration of 0.029 mmol/L^64^. Figure 3 illustrates the effect of different SnO contents in mesoporous SnO-TiO_2_ nanocomposites on the degradation of imazapyr over time. All SnO-TiO_2_ nanocomposites show the ability to significantly reduce imazapyr concentrations, with the 0.1% SnO content consistently achieving the lowest concentrations at various time intervals. The reference catalyst, UV-100, also exhibits degradation capabilities but generally has higher imazapyr concentrations compared to the 0.1% SnO nanocomposite. These results suggest that SnO-TiO_2_ nanocomposites possess photocatalytic activity in degrading imazapyr, and the presence of SnO content significantly enhances their efficiency. Longer illumination times lead to lower imazapyr concentrations, indicating the potential for photocatalytic degradation of this herbicide. Further research may be required to optimize the SnO content and other experimental parameters for the most efficient removal of imazapyr using SnO-TiO_2_ nanocomposites^65^.

Table 3 presents the herbicide residual concentrations over time for different catalyst concentrations (5%, 3%, 1%, 0.5%, 0.1%) and UV100 treatment. Each value represents the mean herbicide concentration with corresponding standard error, illustrating the degradation kinetics under varying conditions. The results demonstrate how catalyst concentration and UV treatment influence the rate of herbicide decomposition over time.

**Table 3:** Time-dependent residual concentration of herbicide (Ct in mmol/L) with various catalyst percentages and UV100 treatment (Mean ± Standard Error).

Min	5%	3%	1%	0.5%	0.1%	UV100
0	0.087 ± 0.003	0.085 ± 0.003	0.084 ± 0.003	0.087 ± 0.003	0.071 ± 0.003	0.083 ± 0.003
15	0.066 ± 0.003	0.053 ± 0.003	0.062 ± 0.003	0.062 ± 0.003	0.052 ± 0.003	0.069 ± 0.003
30	0.058 ± 0.003	0.053 ± 0.003	0.051 ± 0.003	0.05 ± 0.003	0.037 ± 0.002	0.062 ± 0.003
60	0.044 ± 0.002	0.043 ± 0.002	0.041 ± 0.002	0.04 ± 0.002	0.025 ± 0.002	0.058 ± 0.003
90	0.034 ± 0.002	0.033 ± 0.002	0.03 ± 0.002	0.03 ± 0.002	0.017 ± 0.002	0.051 ± 0.003
120	0.025 ± 0.002	0.024 ± 0.002	0.02 ± 0.002	0.021 ± 0.002	0.016 ± 0.002	0.041 ± 0.002
180	0.012 ± 0.002	0.011 ± 0.002	0.019 ± 0.002	0.019 ± 0.002	0.012 ± 0.002	0.029 ± 0.002

#### Mesoporous Ta2O3-TiO2 nanocomposites

**Fig. 4:**
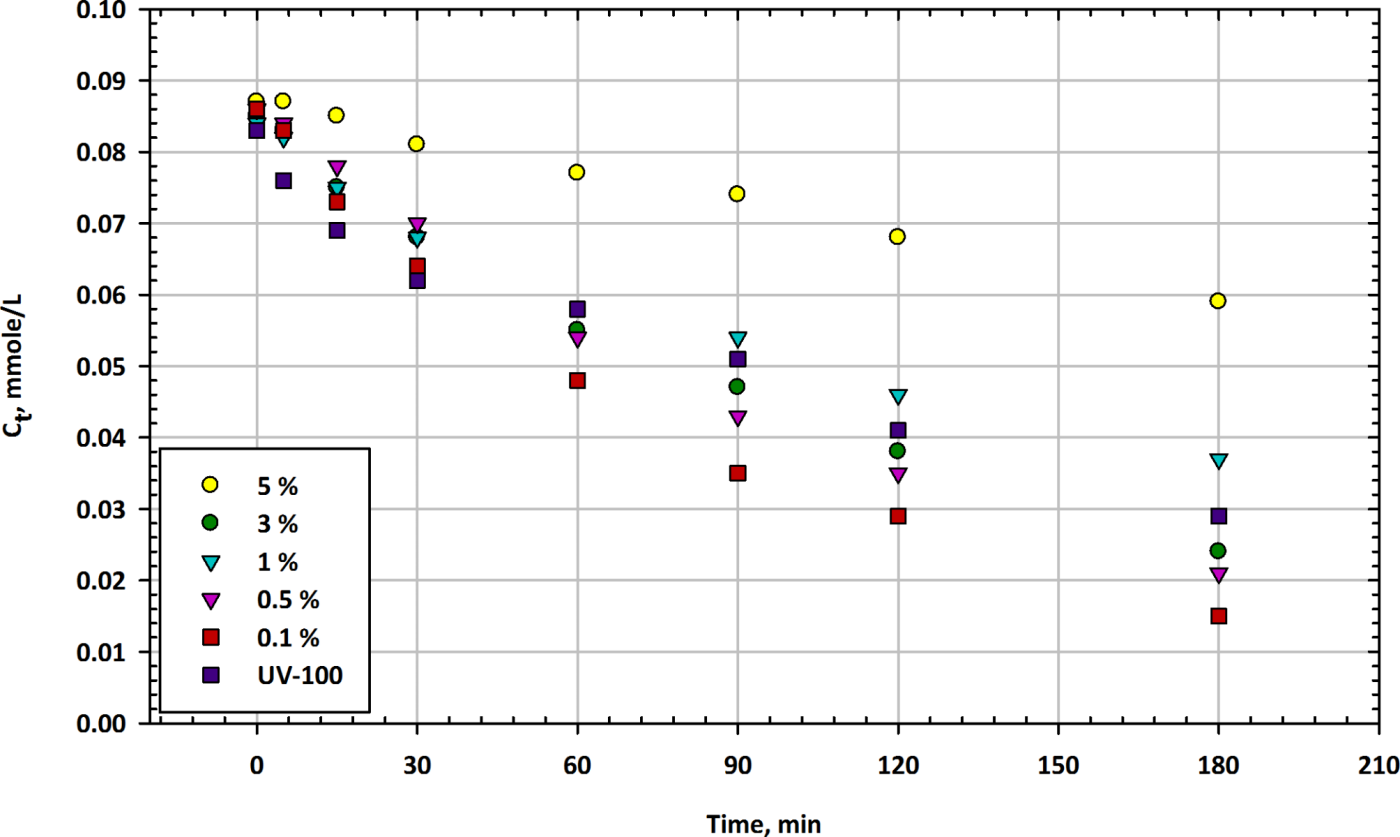
Change in imazapyr concentration as a function of illumination time in the presence of mesoporous Ta_2_O_3_-TiO_2_ nanocomposites at different Ta_2_O_3_ contents compared with Hombikat UV-100 as a reference.

Figure 4 presents the changes in imazapyr concentration (C_t_) as a function of illumination time in the presence of mesoporous Ta_2_O_3_-TiO_2_ nanocomposites at different Ta_2_O_3_ contents, along with Hombikat UV-100 as a reference catalyst. Figure 4 shows the residual concentration of the herbicide at various time intervals. At the start of the experiment, the initial concentration of imazapyr in the solution is approximately 0.087 mmol/L when no catalyst is present. In the presence of Ta_2_O_3_-TiO_2_ nanocomposites, the initial concentrations vary with different Ta_2_O_3_ contents, ranging from 0.084 mmol/L to 0.086 mmol/L. The reference catalyst, UV-100, shows an initial concentration of 0.083 mmol/L. After just 5 min of illumination, the imazapyr concentrations remain relatively constant for all catalysts, indicating limited degradation during this short time. At the 15-minute mark, imazapyr concentrations start to decrease for all catalysts, with the 0.1% Ta_2_O_3_ content achieving the lowest concentration at 0.069 mmol/L^66^. The reference UV-100 catalyst also reduces the concentration to 0.073 mmol/L. After 30 min, imazapyr concentrations continue to decrease for all catalysts, with the 0.1% Ta_2_O_3_ content exhibiting the lowest concentration at 0.062 mmol/L. The reference UV-100 catalyst achieves a concentration of 0.064 mmol/L. At the 60-minute mark, imazapyr concentrations continue to decrease, with the 0.1% Ta_2_O_3_ content achieving the lowest concentration at 0.048 mmol/L. The reference UV-100 catalyst achieves a concentration of 0.058 mmol/L. At 90 min, imazapyr concentrations further decreased for all catalysts, with the 0.1% Ta_2_O_3_ content having the lowest concentration at 0.035 mmol/L. The reference UV-100 catalyst achieves a concentration of 0.051 mmol/L. After 120 min, imazapyr concentrations continue to decrease, with the 0.1% Ta_2_O_3_ content achieving the lowest concentration at 0.029 mmol/L. The reference UV-100 catalyst achieves a concentration of 0.041 mmol/L. At the end of the 180-minute test, imazapyr concentrations continue to decrease for all catalysts, with the 0.1% Ta_2_O_3_ content having the lowest concentration at 0.015 mmol/L. The reference UV-100 catalyst achieves a concentration of 0.029 mmol/L^67^. Figure 4 provides insights into the impact of different Ta_2_O_3_ contents in mesoporous Ta_2_O_3_-TiO_2_ nanocomposites on the degradation of imazapyr over time. Initially, there is limited degradation during the first 5 min for all catalysts, but after 15 min, imazapyr concentrations start to decrease significantly. Among the Ta_2_O_3_-TiO_2_ nanocomposites, the 0.1% Ta_2_O_3_ content consistently achieves the lowest imazapyr concentrations at various time intervals. The reference catalyst, UV-100, also exhibits degradation capabilities but tends to have slightly higher imazapyr concentrations compared to the 0.1% Ta_2_O_3_ nanocomposite. These results suggest that Ta_2_O_3_-TiO_2_ nanocomposites possess photocatalytic activity in degrading imazapyr, and the presence of Ta_2_O_3_ content significantly enhances their efficiency. Longer illumination times lead to lower imazapyr concentrations, indicating the potential for photocatalytic degradation of this herbicide. Further research may be necessary to optimize the Ta_2_O_3_ content and other experimental parameters to maximize the efficiency of Ta_2_O_3_-TiO_2_ nanocomposites for imazapyr removal applications^68^.

Table 4 presents the change in optical density of imazapyr concentrations over various illumination times (0 to 180 min) when exposed to mesoporous Ta_2_O_3_-TiO_2_ nanocomposites with differing Ta_2_O_3_ contents. The data shows a comparative analysis against Hombikat UV-100 as a reference, highlighting the influence of Ta_2_O_3_ concentration on the photostability of imazapyr. Optical density values decrease with increased illumination time, indicating a potential degradation of imazapyr under UV exposure.

**Table 4:** Change in optical density of imazapyr concentrations over illumination time with mesoporous Ta_2_O_3_-TiO_2_ nanocomposites at varying Ta_2_O_3_ contents, compared to hombikat UV-100 as a reference.

Min	100%	5%	1%	0.5%	0.1%	UV100
0	0.087 ± 0.003	0.085 ± 0.003	0.084 ± 0.003	0.086 ± 0.003	0.086 ± 0.003	0.083 ± 0.003
5	0.087 ± 0.003	0.083 ± 0.003	0.082 ± 0.003	0.084 ± 0.003	0.083 ± 0.003	0.076 ± 0.003
15	0.085 ± 0.003	0.075 ± 0.003	0.075 ± 0.003	0.078 ± 0.003	0.073 ± 0.003	0.069 ± 0.003
30	0.081 ± 0.003	0.068 ± 0.003	0.068 ± 0.003	0.070 ± 0.003	0.064 ± 0.003	0.062 ± 0.003
60	0.077 ± 0.003	0.055 ± 0.003	0.054 ± 0.003	0.054 ± 0.003	0.048 ± 0.002	0.058 ± 0.003
90	0.074 ± 0.003	0.047 ± 0.002	0.054 ± 0.003	0.043 ± 0.002	0.035 ± 0.002	0.051 ± 0.003
120	0.068 ± 0.003	0.038 ± 0.002	0.046 ± 0.003	0.035 ± 0.002	0.029 ± 0.002	0.041 ± 0.002
180	0.059 ± 0.003	0.024 ± 0.002	0.037 ± 0.002	0.021 ± 0.002	0.015 ± 0.002	0.029 ± 0.002

#### Mesoporous ZnO-TiO2 nanocomposites

**Fig. 5:**
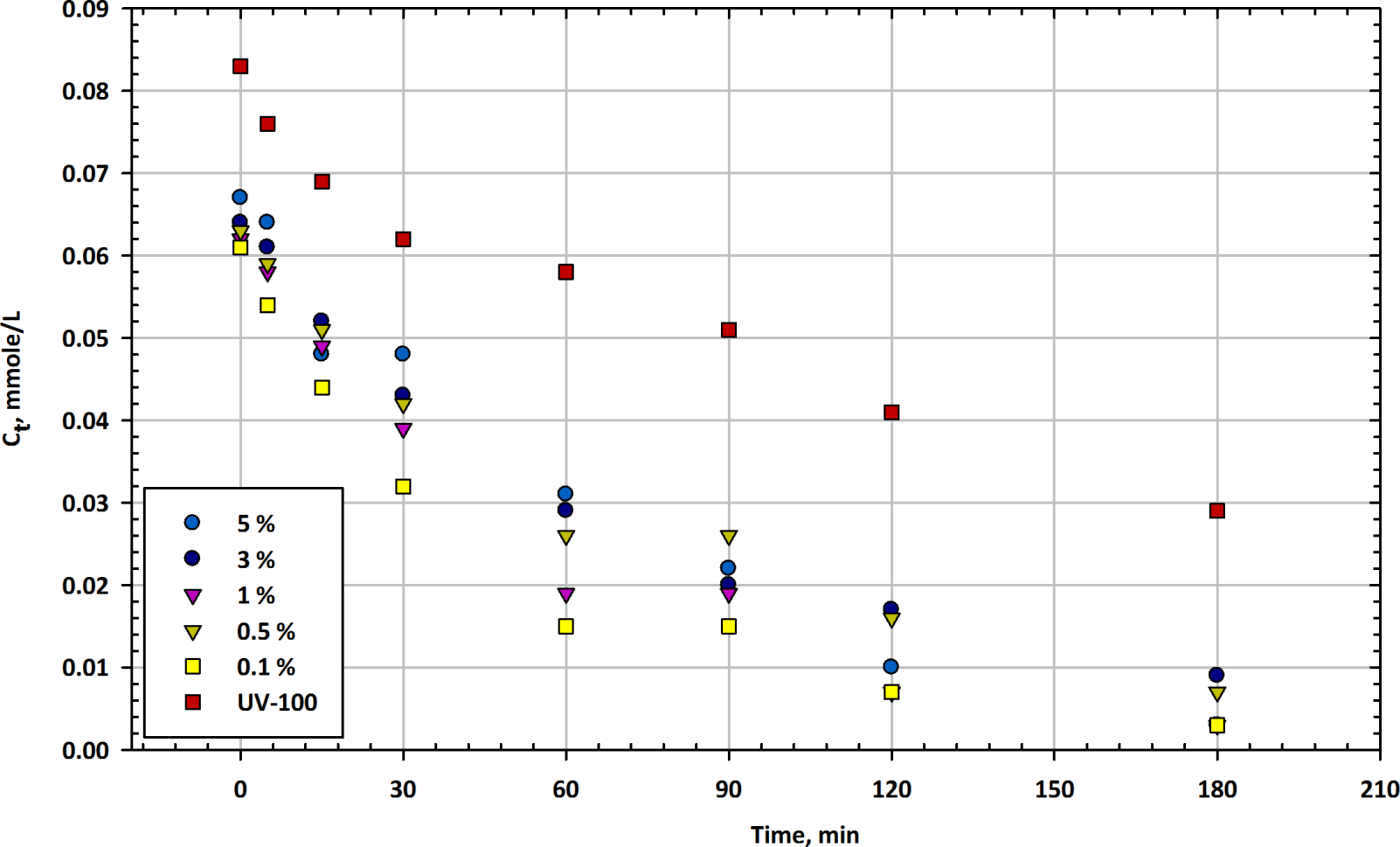
Change in imazapyr concentration as a function of illumination time in the presence of mesoporous ZnO-TiO_2_ nanocomposites at different ZnO contents compared with Hombikat UV-100 as a reference.

Figure 5 presents the changes in imazapyr concentration (C_t_) as a function of illumination time in the presence of mesoporous ZnO-TiO_2_ nanocomposites at different ZnO contents, along with Hombikat UV-100 as a reference catalyst. The table shows the residual concentration of the herbicide at various time intervals. At the beginning of the experiment, the initial concentration of imazapyr in the solution is approximately 0.067 mmol/L when no catalyst is present. In the presence of ZnO-TiO_2_ nanocomposites, the initial concentrations vary with different ZnO contents, ranging from 0.061 mmol/L to 0.064 mmol/L. The reference catalyst, UV-100, shows an initial concentration of 0.083 mmol/L. After 5 min of illumination, all catalysts, including the reference UV-100, show a slight decrease in imazapyr concentrations. However, the reductions are relatively small at this early stage. At the 15-minute mark, imazapyr concentrations begin to decrease more noticeably for all catalysts. Among the ZnO-TiO_2_ nanocomposites, the 0.1% ZnO content achieves the lowest concentration at 0.044 mmol/L. The reference UV-100 catalyst also reduces the concentration to 0.069 mmol/L. After 30 min, imazapyr concentrations continue to decrease for all catalysts, with the 0.1% ZnO content exhibiting the lowest concentration at 0.032 mmol/L^69^. The reference UV-100 catalyst achieves a concentration of 0.062 mmol/L. At the 60-minute mark, imazapyr concentrations further decreased, with the 0.1% ZnO content achieving the lowest concentration at 0.015 mmol/L. The reference UV-100 catalyst achieves a concentration of 0.058 mmol/L. At 90 min, imazapyr concentrations continue to decrease for all catalysts, with the 0.1% ZnO content having the lowest concentration at 0.015 mmol/L. The reference UV-100 catalyst achieves a concentration of 0.051 mmol/L. After 120 min, imazapyr concentrations continue to decrease, with the 0.1% ZnO content achieving the lowest concentration at 0.007 mmol/L. The reference UV-100 catalyst achieves a concentration of 0.041 mmol/L. At the end of the 180-minute test, imazapyr concentrations continue to decrease for all catalysts, with the 0.1% ZnO content having the lowest concentration at 0.003 mmol/L. The reference UV-100 catalyst achieves a concentration of 0.029 mmol/L. Figure 5 provides insights into the impact of different ZnO contents in mesoporous ZnO-TiO_2_ nanocomposites on the degradation of imazapyr over time. While there is a slight reduction in imazapyr concentration during the initial 5 min, significant degradation begins to occur after 15 min of illumination. Among the ZnO-TiO_2_ nanocomposites, the 0.1% ZnO content consistently achieves the lowest imazapyr concentrations at various time intervals^70^. The reference catalyst, UV-100, also exhibits degradation capabilities but generally has slightly higher imazapyr concentrations compared to the 0.1% ZnO nanocomposite. These results suggest that ZnO-TiO_2_ nanocomposites possess photocatalytic activity in degrading imazapyr, and the presence of ZnO content significantly enhances their efficiency. Longer illumination times lead to lower imazapyr concentrations, indicating the potential for photocatalytic degradation of this herbicide. Further research may be necessary to optimize the ZnO content and other experimental parameters to maximize the efficiency of ZnO-TiO_2_ nanocomposites for imazapyr removal applications^71^.

Table 5 illustrates the change in residual imazapyr concentration over time (0 to 180 min) when treated with mesoporous ZnO-TiO_2_ nanocomposites at varying ZnO contents. The data is compared to Hombikat UV-100 as a reference, showing a decline in imazapyr concentration with increased illumination time. The results suggest that the presence of different ZnO contents in the nanocomposites influences the degradation of imazapyr, indicating their potential effectiveness in photodegradation applications.

**Table 5:** Change in residual imazapyr concentration as a function of illumination time with mesoporous ZnO-TiO_2_ nanocomposites at different ZnO contents, compared to hombikat UV-100 as a reference.

Min	100%	5%	1%	0.5%	0.1%	UV100
0	0.067 ± 0.003	0.064 ± 0.003	0.062 ± 0.003	0.063 ± 0.003	0.061 ± 0.003	0.083 ± 0.003
5	0.064 ± 0.003	0.061 ± 0.003	0.058 ± 0.003	0.059 ± 0.003	0.054 ± 0.003	0.076 ± 0.003
15	0.048 ± 0.003	0.052 ± 0.003	0.049 ± 0.003	0.051 ± 0.003	0.044 ± 0.003	0.069 ± 0.003
30	0.048 ± 0.003	0.043 ± 0.003	0.039 ± 0.003	0.042 ± 0.003	0.032 ± 0.003	0.062 ± 0.003
60	0.031 ± 0.003	0.029 ± 0.003	0.019 ± 0.003	0.026 ± 0.003	0.015 ± 0.003	0.058 ± 0.003
90	0.022 ± 0.003	0.02 ± 0.003	0.019 ± 0.003	0.026 ± 0.003	0.015 ± 0.003	0.051 ± 0.003
120	0.01 ± 0.003	0.017 ± 0.003	0.007 ± 0.003	0.016 ± 0.003	0.007 ± 0.003	0.041 ± 0.003
180	0.003 ± 0.003	0.009 ± 0.003	0.003 ± 0.003	0.007 ± 0.003	0.003 ± 0.003	0.029 ± 0.003

#### Mesoporous ZrO-TiO2 nanocomposites

**Fig. 6:**
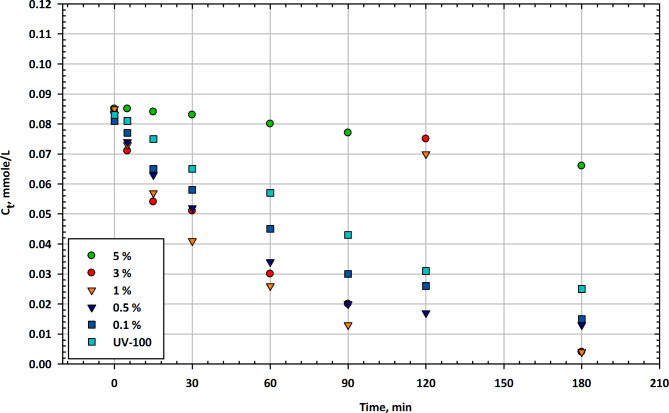
Change in imazapyr concentration as a function of illumination time in the presence of mesoporous ZrO-TiO_2_ nanocomposites at different ZrO contents compared with Hombikat UV-100 as a reference.

Figure 6 presents the changes in imazapyr concentration (C_t_) as a function of illumination time in the presence of mesoporous ZrO-TiO_2_ nanocomposites at different ZrO contents, along with Hombikat UV-100 as a reference catalyst. The table shows the residual concentration of the herbicide at various time intervals. At the start of the experiment, the initial concentration of imazapyr in the solution is approximately 0.085 mmol/L when no catalyst is present. In the presence of ZrO-TiO_2_ nanocomposites, the initial concentrations vary with different ZrO contents, ranging from 0.081 mmol/L to 0.085 mmol/L. The reference catalyst, UV-100, shows an initial concentration of 0.083 mmol/L. After 5 min of illumination, all catalysts, including the reference UV-100, show little to no change in imazapyr concentrations, indicating limited degradation during this short time. At the 15-minute mark, imazapyr concentrations start to decrease for all catalysts. Among the ZrO-TiO_2_ nanocomposites, the 0.1% ZrO content achieves the lowest concentration at 0.065 mmol/L^72^. The reference UV-100 catalyst also reduces the concentration to 0.075 mmol/L. After 30 min, imazapyr concentrations continue to decrease for all catalysts, with the 0.1% ZrO content exhibiting the lowest concentration at 0.041 mmol/L. The reference UV-100 catalyst achieves a concentration of 0.052 mmol/L. At the 60-minute mark, imazapyr concentrations further decreased, with the 0.1% ZrO content achieving the lowest concentration at 0.026 mmol/L. The reference UV-100 catalyst achieves a concentration of 0.045 mmol/L^73^. At 90 min, imazapyr concentrations continue to decrease for all catalysts, with the 0.1% ZrO content having the lowest concentration at 0.013 mmol/L. The reference UV-100 catalyst achieves a concentration of 0.030 mmol/L. After 120 min, imazapyr concentrations continue to decrease, with the 0.1% ZrO content achieving the lowest concentration at 0.017 mmol/L. The reference UV-100 catalyst achieves a concentration of 0.031 mmol/L. At the end of the 180-minute test, imazapyr concentrations continue to decrease for all catalysts, with the 0.1% ZrO content having the lowest concentration at 0.004 mmol/L. The reference UV-100 catalyst achieves a concentration of 0.025 mmol/L. Figure 6 provides insights into the impact of different ZrO contents in mesoporous ZrO-TiO_2_ nanocomposites on the degradation of imazapyr over time^74^. Initially, there is limited degradation during the first 5 min for all catalysts. Significant degradation starts to occur after 15 min of illumination. Among the ZrO-TiO_2_ nanocomposites, the 0.1% ZrO content consistently achieves the lowest imazapyr concentrations at various time intervals. The reference catalyst, UV-100, also exhibits degradation capabilities but generally has slightly higher imazapyr concentrations compared to the 0.1% ZrO nanocomposite. These results suggest that ZrO-TiO_2_ nanocomposites possess photocatalytic activity in degrading imazapyr, and the presence of ZrO content significantly enhances their efficiency. Longer illumination times lead to lower imazapyr concentrations, indicating the potential for photocatalytic degradation of this herbicide. Further research may be necessary to optimize the ZrO content and other experimental parameters to maximize the efficiency of ZrO-TiO_2_ nanocomposites for imazapyr removal applications^75^.

Table 6 presents the residual concentrations of imazapyr over varying illumination times with mesoporous ZrO-TiO_2_ nanocomposites at different ZrO contents. The data illustrate the degradation of imazapyr, showcasing a comparative analysis with Hombikat UV-100 as a reference. As illumination time increases, a notable decrease in imazapyr concentration is observed across all ZrO content levels, highlighting the efficacy of the mesoporous nanocomposites in facilitating herbicide degradation.

**Table 6:** Change in residual imazapyr concentration over illumination time with mesoporous ZrO-TiO_2_ nanocomposites at various ZrO contents: a comparative analysis with hombikat UV-100 as a reference.

Min	100%	1%	0.6%	0.2%	0.1%	0.02
0	0.085 ± 0.003	0.084 ± 0.003	0.085 ± 0.003	0.083 ± 0.003	0.081 ± 0.003	0.083 ± 0.003
5	0.085 ± 0.003	0.071 ± 0.003	0.073 ± 0.003	0.074 ± 0.003	0.077 ± 0.003	0.081 ± 0.003
15	0.084 ± 0.003	0.054 ± 0.003	0.057 ± 0.003	0.063 ± 0.003	0.065 ± 0.003	0.075 ± 0.003
30	0.083 ± 0.003	0.051 ± 0.003	0.041 ± 0.003	0.052 ± 0.003	0.058 ± 0.003	0.065 ± 0.003
60	0.08 ± 0.003	0.03 ± 0.003	0.026 ± 0.003	0.034 ± 0.003	0.045 ± 0.003	0.057 ± 0.003
90	0.077 ± 0.003	0.020 ± 0.003	0.013 ± 0.003	0.02 ± 0.003	0.03 ± 0.003	0.043 ± 0.003
120	0.075 ± 0.003	0.075 ± 0.003	0.07 ± 0.003	0.017 ± 0.003	0.026 ± 0.003	0.031 ± 0.003
180	0.066 ± 0.003	0.004 ± 0.003	0.004 ± 0.003	0.013 ± 0.003	0.015 ± 0.003	0.025 ± 0.003

### Comparative results of mesoporous nanocomposites

**Fig. 7:**
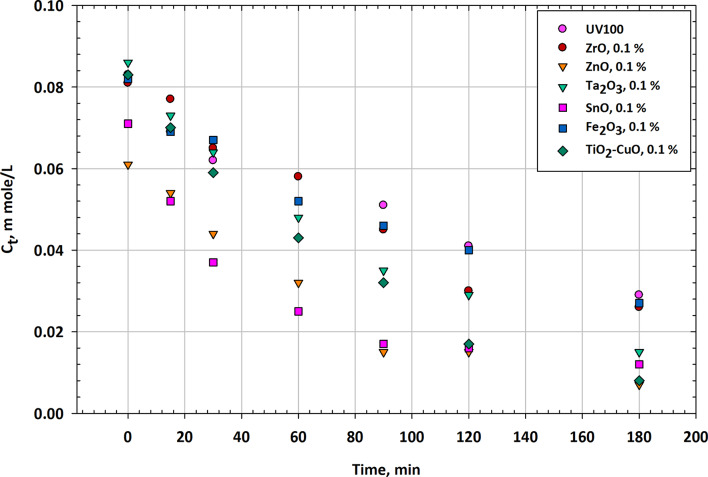
Photodegradation efficiency of imazapyr: a comparative analysis of different composite catalysts with 0.1 wt% metal oxide additives.

Figure 7 provides a comparison between different composite catalysts for the photodegradation of the herbicide Imazapyr at a concentration of 0.1% of the metal oxide in each composite. The table displays the residual concentrations (Ct) at various illumination times and includes TiO_2_-CuO, Fe_2_O_3_, SnO, Ta_2_O_3_, ZnO, ZrO, and UV100 as reference catalysts. At the start of the experiment (0 min), all catalysts exhibit varying initial concentrations of imazapyr^76^. Among the composite catalysts, TiO_2_-CuO, Fe_2_O_3_, and Ta_2_O_3_ have initial concentrations slightly higher than that of UV100, while SnO, ZnO, and ZrO have lower initial concentrations. Notably, CuO-TiO_2_ (TiO_2_-CuO) has the highest initial concentration at 0.083 mmol/L, while ZnO-TiO_2_ (ZnO) has the lowest at 0.061 mmol/L. After 15 min of illumination, all composite catalysts show a reduction in imazapyr concentration. CuO-TiO_2_ (TiO_2_-CuO) and Fe_2_O_3_ exhibit similar reductions and have the lowest concentrations at 0.07 mmol/L and 0.069 mmol/L, respectively. Among all catalysts, SnO-TiO_2_ (SnO) has the lowest concentration at 0.052 mmol/L. ZrO-TiO_2_ (ZrO) shows the least reduction, with a concentration of 0.077 mmol/L. At 30 min, imazapyr concentrations continue to decrease for all catalysts. SnO-TiO_2_ (SnO) has the lowest concentration at 0.037 mmol/L, while ZrO-TiO_2_ (ZrO) still exhibits slower degradation with a concentration of 0.065 mmol/L. After 60 min, imazapyr concentrations continue to decline. SnO-TiO_2_ (SnO) and ZnO-TiO_2_ (ZnO) have the lowest concentrations at 0.025 mmol/L and 0.032 mmol/L, respectively^77^. ZrO-TiO_2_ (ZrO) remains the slowest in degrading imazapyr with a concentration of 0.058 mmol/L. At 90 min, the imazapyr concentrations continue to decrease. SnO-TiO_2_ (SnO) still exhibits efficient degradation with a concentration of 0.015 mmol/L, while ZrO-TiO_2_ (ZrO) lags with a concentration of 0.03 mmol/L. After 120 min, imazapyr concentrations continue to decrease for all catalysts. SnO-TiO_2_ (SnO) and ZnO-TiO_2_ (ZnO) achieve lower concentrations compared to other catalysts. At the end of the 180-minute test, imazapyr concentrations further decreased for all catalysts. SnO-TiO_2_ (SnO) has the lowest concentration at 0.012 mmol/L, indicating efficient degradation. ZrO-TiO_2_ (ZrO) still has a relatively higher concentration at 0.026 mmol/L, indicating slower degradation. Figure 7 provides a comparative view of the efficiency of different composite catalysts in the photodegradation of Imazapyr. SnO-TiO_2_ (SnO) consistently performs well, achieving the lowest concentrations at most time intervals, indicating its strong photocatalytic activity for Imazapyr degradation. TiO_2_-CuO (CuO-TiO_2_) and Fe_2_O_3_ exhibit competitive degradation efficiency with Imazapyr concentrations close to SnO-TiO_2_. ZnO-TiO_2_ (ZnO) shows relatively efficient degradation^78^. ZrO-TiO_2_ (ZrO) appears to be less effective in degrading Imazapyr compared to other composites, with slower degradation rates and higher residual concentrations. The reference catalyst, UV100, exhibits moderate degradation capabilities but tends to have slightly higher imazapyr concentrations compared to some of the composite catalysts. The choice of composite catalyst plays a crucial role in the photodegradation of Imazapyr, and further research may be needed to optimize the conditions for each catalyst to maximize their efficiency in herbicide removal applications^79^.

The superior performance of CuO-TiO_2_ over Fe_2_O_3_-TiO_2_ in many photocatalytic and corrosion inhibition applications can be attributed to several key factors related to their electronic structures, band alignment, charge separation efficiency, and photocatalytic activity. CuO-TiO_2_: CuO has a relatively narrow band gap (~ 1.2–1.7 eV), which allows it to absorb a significant portion of the visible light spectrum. When combined with TiO_2_ (band gap ~ 3.2 eV), CuO extends TiO_2_’s light absorption capabilities from UV to visible light. This enables CuO-TiO_2_ composites to be more active under sunlight or visible light, making them highly efficient for applications such as photocatalysis and environmental remediation. CuO-TiO_2_: In a CuO-TiO_2_ heterojunction, the conduction band (CB) of CuO is lower than that of TiO_2_, while the valence band (VB) of CuO is higher than TiO_2_. This favorable band alignment facilitates the transfer of photogenerated electrons from the conduction band of TiO_2_ to the conduction band of CuO, while the holes remain in the valence band of TiO_2_. This separation of electrons and holes reduces recombination, increasing photocatalytic efficiency. CuO acts as an electron sink due to its lower conduction band, allowing it to effectively trap and transfer electrons. This greatly enhances charge separation, reducing electron-hole recombination, and improving photocatalytic performance. The Schottky barrier formed at the CuO-TiO_2_ interface effectively suppresses electron-hole recombination. This barrier arises due to the difference in Fermi levels between CuO and TiO_2_, which prevents the backflow of electrons into TiO_2_, keeping the electrons and holes separated for a longer period and allowing them to participate in redox reactions. Plasmonic Enhancement: In some cases, CuO can exhibit plasmonic effects that enhance local electromagnetic fields and improve charge separation even further. CuO has a high redox potential and the ability to undergo reversible redox reactions (Cu^2+^ ↔ Cu⁺), which makes it highly effective for photocatalytic and electrochemical processes. The Cu^2+^/Cu⁺ redox cycle contributes to CuO’s superior ability to catalyze oxidation-reduction reactions, such as the degradation of pollutants or water splitting. Photocatalytic Degradation: CuO-TiO_2_ composites are particularly effective in photocatalytic degradation of organic pollutants and hydrogen production due to CuO’s higher catalytic efficiency in redox reactions. CuO is relatively more stable in various environmental conditions compared to Fe_2_O_3_. CuO-TiO_2_ composites tend to have higher durability, especially in harsh environments such as strong acidic or alkaline media, where Fe_2_O_3_ may undergo dissolution or degradation. Longer Operational Life: The stability of CuO in photocatalytic and corrosion inhibition applications makes it a preferred choice for long-term operation. CuO-TiO_2_ composites often exhibit higher photocurrent densities compared to Fe_2_O_3_-TiO_2_. This is due to the more efficient charge separation and electron transfer processes in CuO-TiO_2_ systems, leading to higher overall photocatalytic efficiency. Faster Electron Transfer: The high electron mobility in CuO contributes to faster charge transfer between CuO and TiO_2_, resulting in enhanced photocurrent generation and more efficient energy conversion. These factors collectively explain why CuO-TiO_2_ typically outperforms Fe_2_O_3_-TiO_2_ in various applications, including photocatalysis, energy conversion, and environmental remediation.

Table 7 presents the residual concentrations of Imazapyr over varying illumination times using different metal oxide composite catalysts. Each catalyst, including TiO_2_-CuO, Fe_2_O_3_, SnO, Ta_2_O_3_, ZnO, and ZrO, demonstrates distinct degradation efficiencies compared to the UV100 reference. The results indicate a general trend of decreasing herbicide concentration with increased illumination time, highlighting the effectiveness of the catalysts in facilitating photodegradation.

**Table 7:** Comparative evaluation of different composite catalysts for the photodegradation of imazapyr herbicide at 0.1% metal oxide concentration.

Min	TiO_2_-CuO, 0.1%	Fe_2_O_3_, 0.1%	SnO, 0.1%	Ta_2_O_3_, 0.1%	ZnO, 0.1%	ZrO, 0.1%	UV100
0	0.083 ± 0.003	0.082 ± 0.003	0.071 ± 0.003	0.086 ± 0.003	0.061 ± 0.003	0.081 ± 0.003	0.083 ± 0.003
15	0.07 ± 0.003	0.069 ± 0.003	0.052 ± 0.003	0.073 ± 0.003	0.054 ± 0.003	0.077 ± 0.003	0.069 ± 0.003
30	0.059 ± 0.003	0.067 ± 0.003	0.037 ± 0.003	0.064 ± 0.003	0.044 ± 0.003	0.065 ± 0.003	0.062 ± 0.003
60	0.043 ± 0.003	0.052 ± 0.003	0.025 ± 0.003	0.048 ± 0.003	0.032 ± 0.003	0.058 ± 0.003	0.058 ± 0.003
90	0.032 ± 0.003	0.046 ± 0.003	0.017 ± 0.003	0.035 ± 0.003	0.015 ± 0.003	0.045 ± 0.003	0.051 ± 0.003
120	0.017 ± 0.003	0.04 ± 0.003	0.016 ± 0.003	0.029 ± 0.003	0.015 ± 0.003	0.03 ± 0.003	0.041 ± 0.003
180	0.008 ± 0.003	0.027 ± 0.003	0.012 ± 0.003	0.015 ± 0.003	0.007 ± 0.003	0.026 ± 0.003	0.029 ± 0.003

### Comparative characterization results

#### Structural analysis by X-ray diffraction (XRD)

**Fig. 8:**
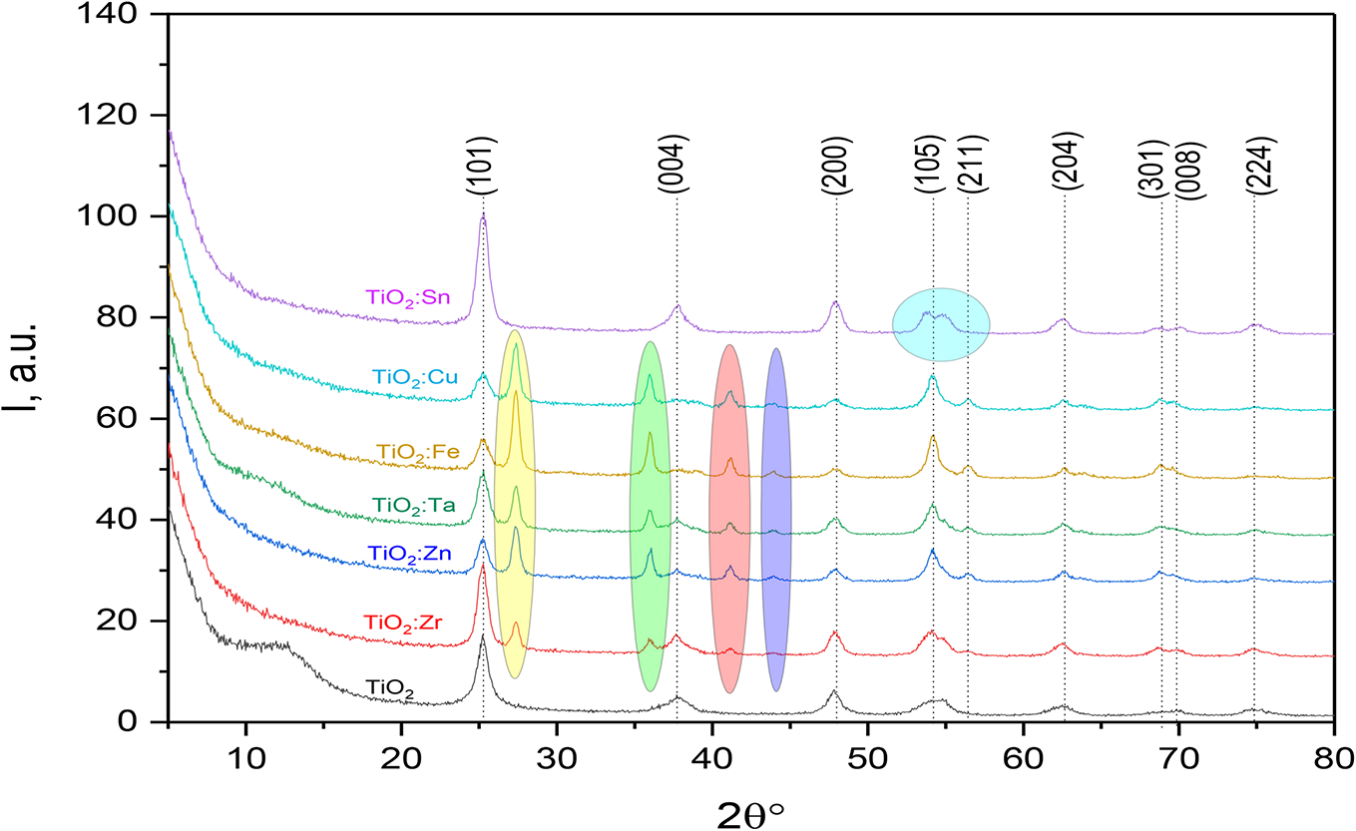
XRD curves of pure TiO_2_ and TiO_2_ doped with Zr, Zn, Ta, Fe, Cu, and Sn.

Using X-ray diffraction analysis, we determined the crystalline phase of TiO_2_ and TiO_2_ doped with various oxide nanoparticles. In Fig. 8, the XRD data for pure TiO_2_ exhibited broad peaks at 2θ values of 25.27, 37.71, 47.98, 54.21, 56.35, 62.55, 68.77, 69.83, and 74.76°, corresponding to planes (101), (004), (200), (105), (211), (204), (301), (008), and (224). This confirms the tetragonal structure of Ti0.72O2 nanoparticles, as indicated by JCPDS reference code 00-086-1157. By doping by different oxides, i.e. Zr, Zn, Ta, Fe, and Cu, 4 new peaks (at 27.20, 35.93, 41.05, and 43.96° highlighted by yellow, green, red, and blue colors, respectively) with different intensity and different shapes. For Zr-doped TiO_2_, the peaks related to orthorhombic Zr_5_Ti_7_O_24_ (JCPDS: 00-084-1019). For Zn-doped TiO_2_, these peaks are related to cubic Zn_2_Ti_3_O_8_ (JCPDS: 00-087-1781). For Ta-doped TiO_2_, the peaks are related to tetragonal TiTaO_4_ (JCPDS: 00-071-0929). For Fe-doped TiO_2_, these peaks are related to hexagonal Fe_2_Ti_3_O_9_ (JCPDS: 00-029-1494). For Cu-doped TiO_2_, these peaks are related to cubic Cu_3_Ti_3_O (JCPDS: 00-075-0400). The Sn-doped TiO_2_ did not show the 4 peaks that were observed in other dopants, but the peak at 54.21 splits into two close peaks at 53.66° and 54.77° (highlighted by turquoise color). These two peaks are related to tetragonal (Ti0.85Sn0.15) O2 (JCPDS: 00-081-1387)^80^.

In Fig. 8, X-ray Diffraction (XRD) curves of pure TiO_2_ and TiO_2_ doped with Zr, Zn, Ta, Fe, Cu, and Sn are presented. The XRD analysis provides crucial information about the crystal structure and phase composition of the synthesized materials. Here’s a detailed discussion of the observed XRD curves^81^. In the case of pure TiO_2_, the XRD pattern of pure TiO_2_ serves as a reference for the undoped material. Key peaks corresponding to the anatase or rutile phases of TiO_2_ are expected and can be identified. The sharpness and position of these peaks indicate the crystallinity and purity of the synthesized TiO_2_. For the TiO_2_/Zr composite, the XRD curve for TiO_2_ doped with Zr is analyzed for any shifts in peak positions or the appearance of new peaks^82^. The presence of ZrO_2_ peaks alongside TiO_2_ peaks suggests the successful incorporation of Zr into the TiO_2_ lattice. In the case of TiO_2_/Zn composite, similar analysis is applied to the TiO_2_/Zn composite. Any additional peaks corresponding to ZnO should be observed. Shifts in TiO_2_ peaks may also indicate interactions between TiO_2_ and ZnO phases. For TiO_2_/Ta composite, the XRD pattern for TiO_2_/Ta is scrutinized for characteristic peaks of Ta_2_O_5_. The coexistence of TiO_2_ and Ta_2_O_5_ peaks confirms the presence of both materials^83^. For TiO_2_/Fe composite, TiO_2_ doped with Fe, changes in peak positions or the emergence of Fe_2_O_3_ peaks are examined. The XRD pattern indicates whether Fe has been successfully incorporated into the TiO_2_ lattice. In TiO_2_/Cu composite, the XRD curve for TiO_2_/Cu is assessed for CuO peaks alongside TiO_2_ peaks. Any shifts or broadening of TiO_2_ peaks may suggest interactions with CuO^84^. Finally, in the case of TiO_2_/Sn composite, the XRD analysis of TiO_2_/Sn focuses on identifying peaks associated with SnO_2_. Confirmation of SnO_2_ presence in the composite is crucial for assessing the doping effect. Generally, changes in peak intensities, positions, or the emergence of new peaks indicate the successful doping of TiO_2_ with the respective elements. The preservation of key TiO_2_ peaks demonstrates the retention of its crystal structure even after doping. Successful doping can lead to alterations in the electronic structure, enhancing the photocatalytic properties of TiO_2_^85^. The nature of the observed phases (anatase, rutile, ZnO, Ta_2_O_5_, Fe_2_O_3_, CuO, SnO_2_) influences the overall properties of the composite materials. Detailed peak assignments and quantitative phase analysis could provide deeper insights into the doping effects^86^. Complementary techniques, such as SEM and TEM, can corroborate the structural information obtained from XRD. In conclusion, the XRD curves in Fig. 8 confirm the successful doping of TiO_2_ with Zr, Zn, Ta, Fe, Cu, and Sn, providing valuable information about the crystallographic changes induced by the dopants. These findings are pivotal for understanding and optimizing the enhanced photocatalytic applications of the doped TiO_2_ composites^87^.

#### **Transmission electron microscopy (TEM) results**

**Fig. 9:**
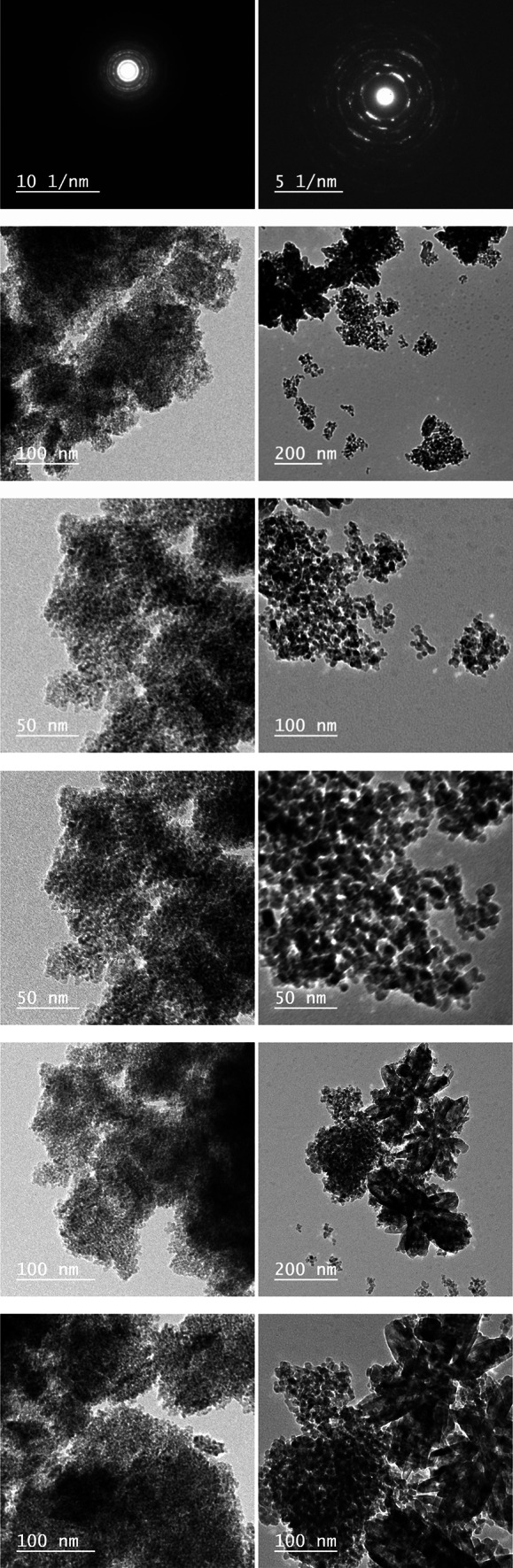
TEM images unveil the morphological insights of pure TiO_2_ and its composite forms with Zr, Zn, Ta, Fe, Cu, and Sn.

The transmission electron microscopy (TEM) images presented in Fig. 9 offer valuable insights into the morphological characteristics of both pure TiO_2_ and its composite forms with Zr, Zn, Ta, Fe, Cu, and Sn. These composite variations are crucial for tailoring the properties of TiO_2_, expanding its potential applications in various fields. In the case of pure TiO_2_, the TEM image of pure TiO_2_ serves as a baseline, showcasing the inherent morphology of titanium dioxide nanoparticles^88^. The characteristic features, such as particle size, shape, and distribution, are essential for understanding the structural properties of TiO_2_. For the TiO_2_-Zr composite, the composite form with Zr exhibits distinct morphological changes compared to pure TiO_2_^89^. The TEM image reveals potential alterations in particle size, agglomeration, or even the introduction of new structures, indicating the influence of Zr on the TiO_2_ matrix. In the case of the TiO_2_-Zn composite, similar to TiO_2_-Zr, the composite with Zn introduces modifications in the morphology^90^. The TEM image highlights any shifts in particle characteristics, shedding light on the synergistic effects of Zn composite with TiO_2_. In TiO_2_-Ta composite, the TEM image of TiO_2_-Ta showcases the impact of tantalum composite on the titanium dioxide structure. Observations related to particle arrangement and size distribution provide crucial information on the composite’s influence^91^. For TiO_2_-Fe composite, composite TiO_2_ with iron (Fe) can impart unique properties. The TEM image reveals any changes in the nanoscale features, helping to understand how the introduction of iron alters the morphology and structure of TiO_2_. The TiO_2_-Cu composite, copper (Cu) composite introduces its own set of characteristics^92^. The TEM image elucidates how the addition of copper influences the TiO_2_ morphology, potentially impacting properties such as conductivity or catalytic activity. But in the case of TiO_2_-Sn composite, Tin (Sn) composite can lead to morphological variations in TiO_2_. The TEM image allows for a detailed examination of these changes, aiding in the assessment of Sn’s role in modifying the TiO_2_ nanostructure^93^. In summary, the TEM images provide a comprehensive visual analysis of the morphological features in each composite form of TiO_2_. These insights are instrumental in understanding the nanoscale changes induced by compositing elements, contributing to the design and optimization of TiO_2_-based materials for diverse applications^94^.

#### Surface area measurements

**Fig. 10:**
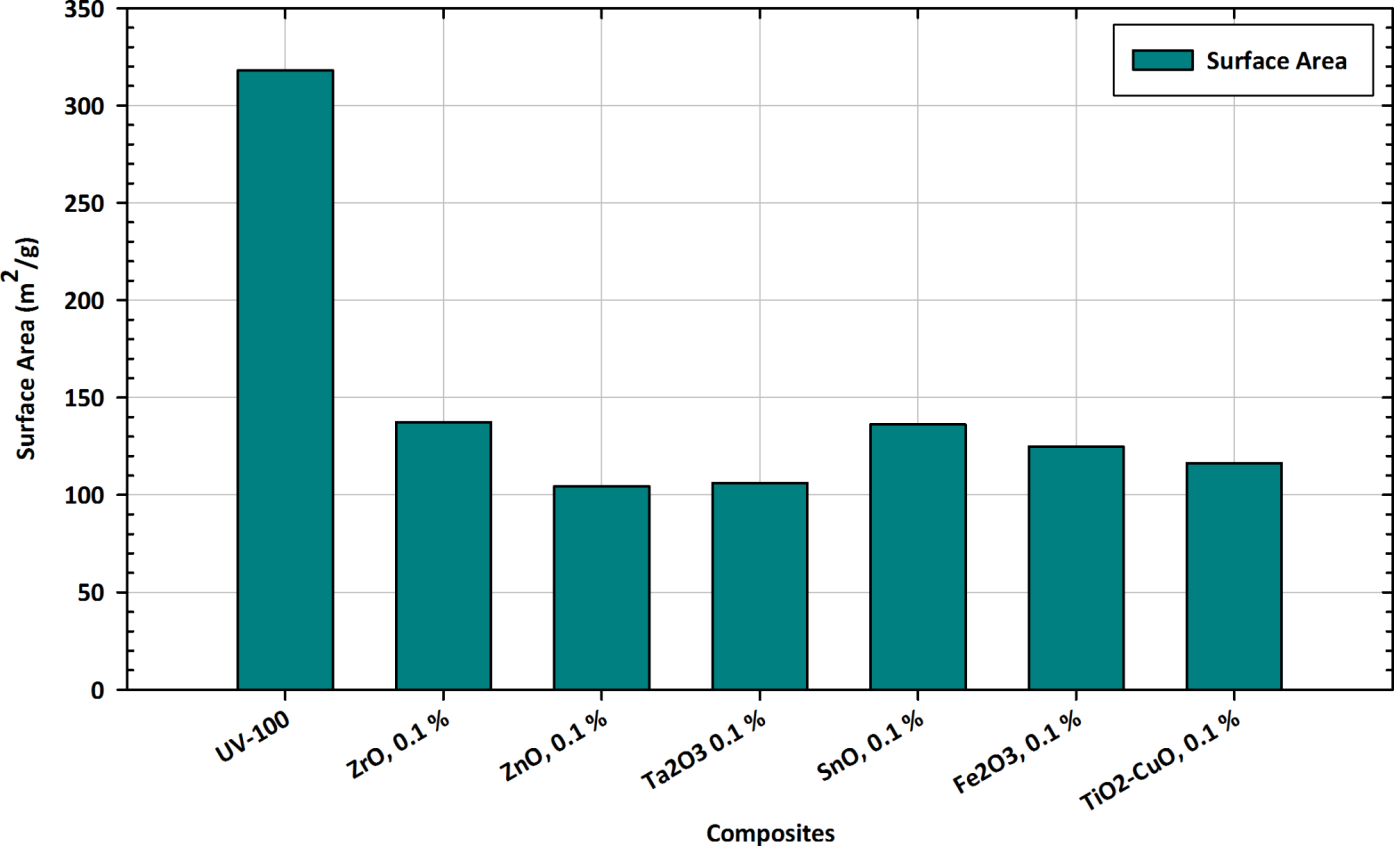
Surface area variations in pure TiO_2_ and composite forms with Zr, Zn, Ta, Fe, Cu, and Sn for tailored applications.

The surface area variations depicted in Fig. 10 provide crucial insights into the tailored applications of pure TiO_2_ and its composite forms with Zr, Zn, Ta, Fe, Cu, and Sn. Each composition exhibits distinct surface area characteristics that hold significant implications for their practical utilization. Pure TiO_2_ demonstrates a baseline surface area of UV-100, serving as a reference for comparison. Alloying with ZrO, ZnO, Ta_2_O_3_, SnO, Fe_2_O_3_, and TiO_2_-CuO at 0.1% concentration results in diverse surface area alterations^95^. Notably, ZrO exhibits a substantial reduction, while ZnO and Ta_2_O_3_ show moderate decreases. In contrast, SnO and Fe_2_O_3_ demonstrate a slight increase in surface area, indicating nuanced impacts. The composite form TiO_2_-CuO at 0.1% concentration stands out with a distinctive surface area value, suggesting a unique interplay between TiO_2_ and CuO. These variations in surface area are pivotal for tailoring the materials to specific applications, influencing factors like catalytic activity, adsorption capacity, and overall performance. The observed trends in surface area variations provide a foundation for optimizing the compositions based on targeted functionalities, contributing to the advancement of tailored applications in diverse fields^96^.

#### Av. Pore size measurements

**Fig. 11:**
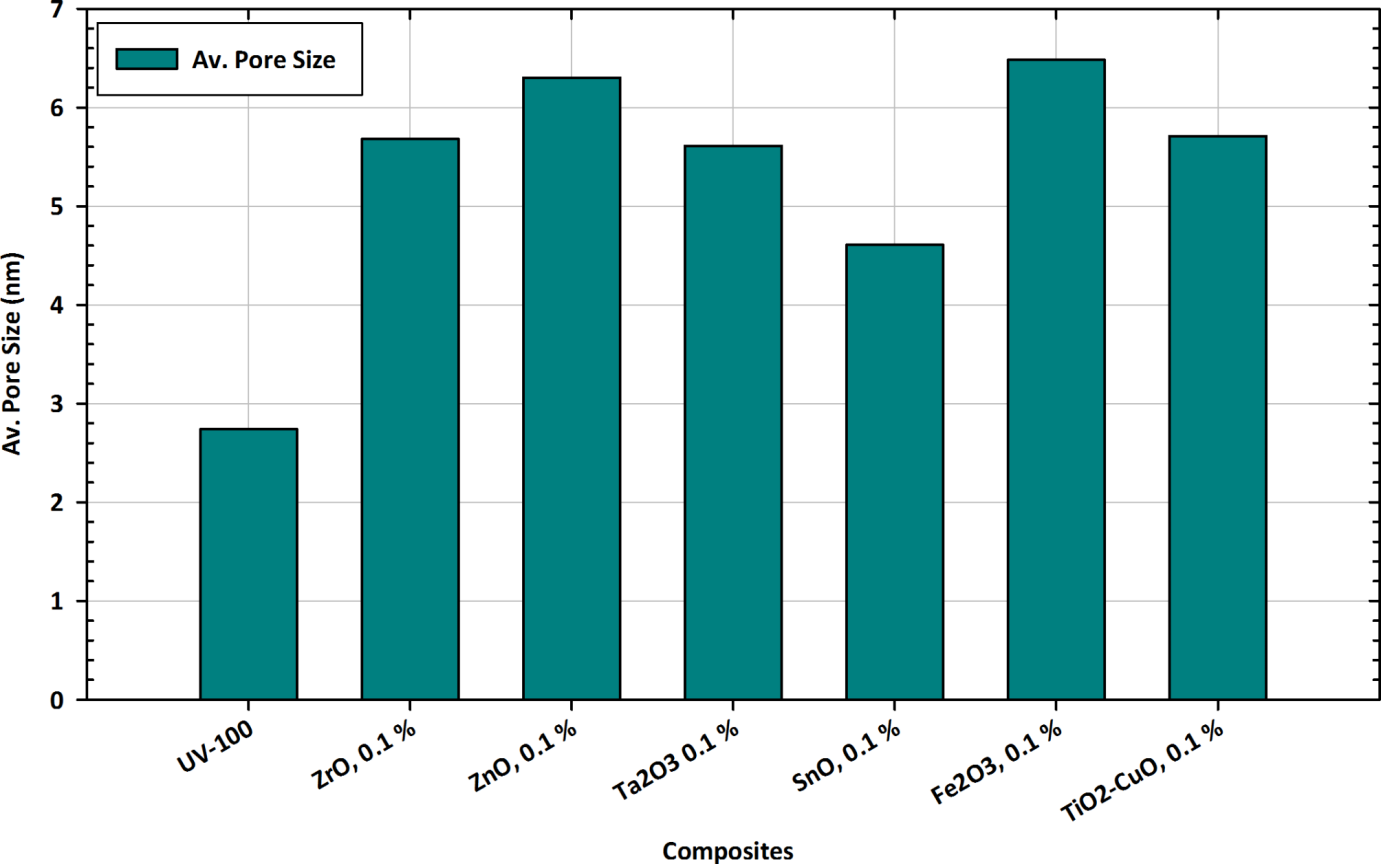
Av. pore size variations in pure TiO_2_ and composite forms with Zr, Zn, Ta, Fe, Cu, and Sn for tailored applications.

The variations in average pore size illustrated in Fig. 11 provide valuable insights into the tailored applications of pure TiO_2_ and its composite forms with Zr, Zn, Ta, Fe, Cu, and Sn. The average pore size is a critical parameter influencing the materials’ porosity, surface reactivity, and potential applications. Pure TiO_2_, represented by UV-100, exhibits an average pore size of 2.7399, serving as a reference point for comparison. Alloying with different elements at a 0.1% concentration results in diverse changes in average pore size^97^. Notably, ZrO shows a significant increase, indicating a substantial alteration in the material’s pore structure. ZnO and Ta_2_O_3_ exhibit moderate increases, while SnO demonstrates a slight decrease in average pore size. Fe_2_O_3_ and TiO_2_-CuO exhibit notable increases, suggesting distinctive impacts on pore characteristics^98^. The composite form TiO_2_-CuO at 0.1% concentration stands out with a notable change in average pore size, indicating a unique interplay between TiO_2_ and CuO. These variations are crucial for tailoring the materials to specific applications, influencing factors like adsorption capacity, diffusion rates, and overall performance. The observed trends in average pore size variations provide essential information for optimizing compositions based on targeted functionalities, contributing to advancements in tailored applications in diverse fields^99^.

#### Particle size measurements

**Fig. 12:**
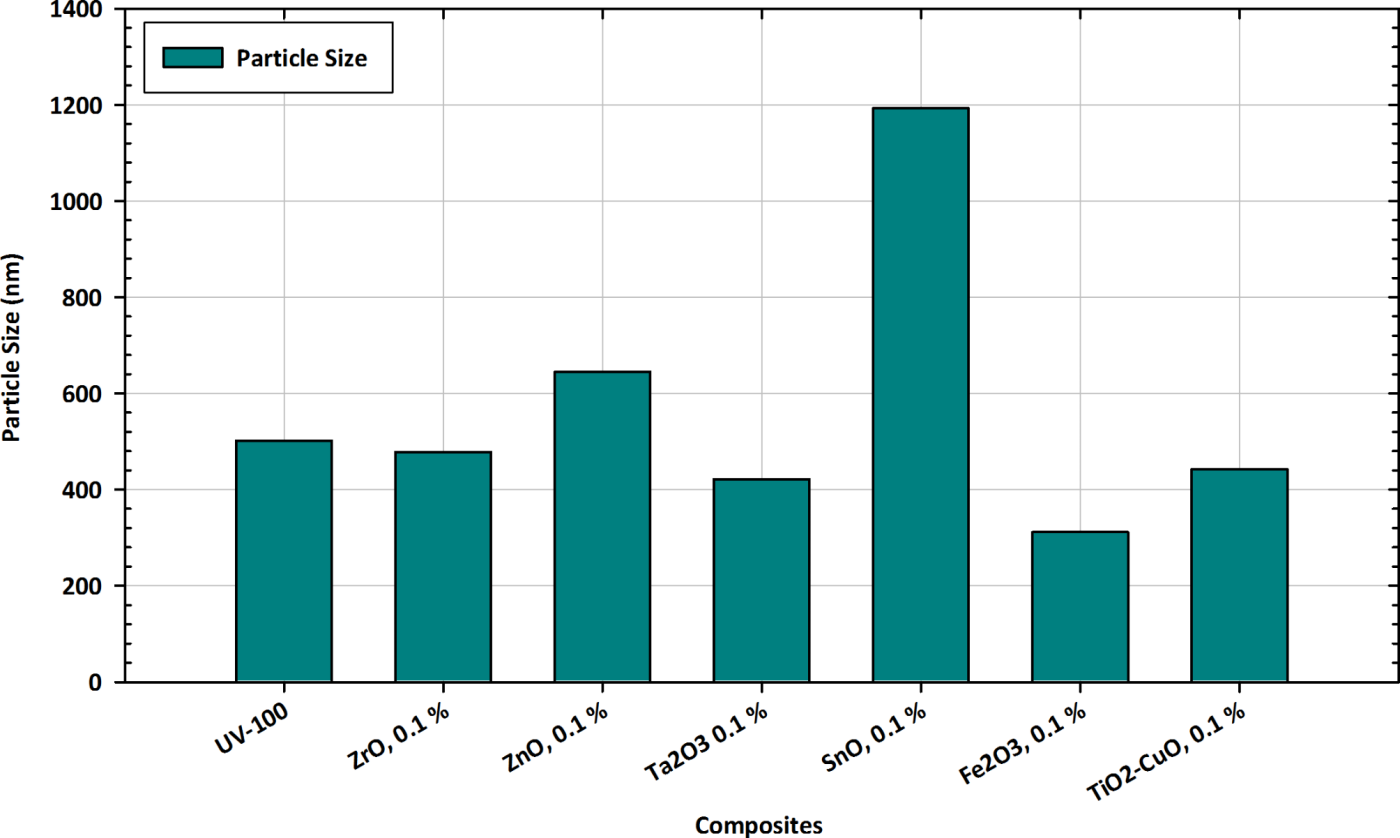
Particle size variations in pure TiO_2_ and composite forms with Zr, Zn, Ta, Fe, Cu, and Sn for tailored applications.

The particle size variations depicted in Fig. 12 offer crucial insights into the tailored applications of pure TiO_2_ and its composite forms with Zr, Zn, Ta, Fe, Cu, and Sn. Particle size is a fundamental characteristic influencing the materials’ reactivity, stability, and applications in various fields. Pure TiO_2_, represented by UV-100, exhibits a particle size of 501.7, serving as a baseline for comparison^100^. Alloying with different elements at a 0.1% concentration results in diverse changes in particle size. Notably, ZnO and TiO_2_-CuO show a decrease in particle size, suggesting a refining effect on the materials’ structure. ZrO and Ta_2_O_3_ exhibit marginal reductions, while Fe_2_O_3_ shows a moderate decrease. SnO, on the other hand, demonstrates a substantial increase in particle size^101^. Among the composite forms, SnO at 0.1% concentration stands out with a significant increase in particle size, indicating a distinctive impact on the overall structure. These variations are crucial for tailoring the materials to specific applications, influencing factors like photocatalytic efficiency, dispersion properties, and overall performance. The observed trends in particle size variations provide essential information for optimizing compositions based on targeted functionalities, contributing to advancements in tailored applications across diverse industries^102^.

#### Variance (P.I.) measurements

**Fig. 13:**
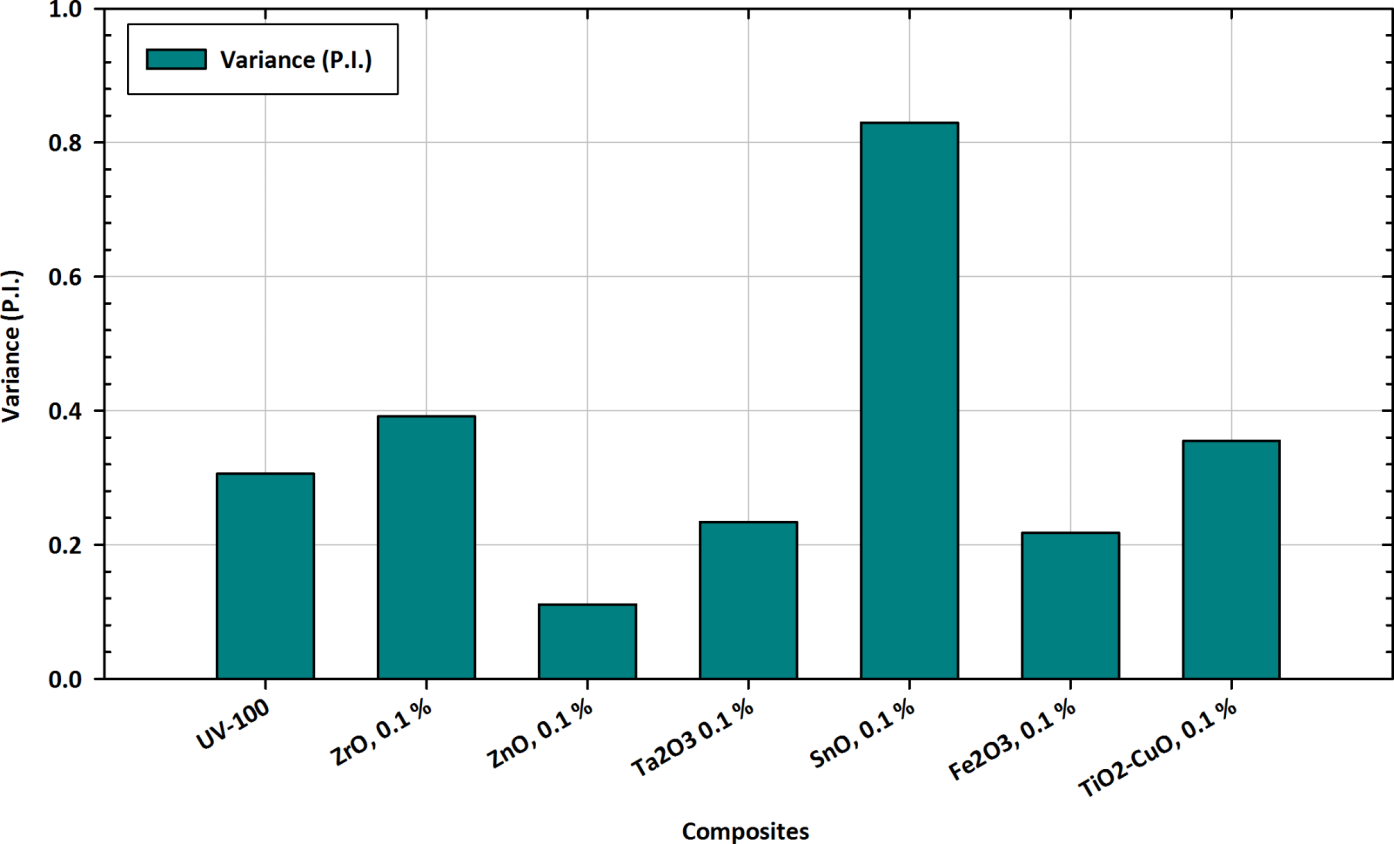
Variance (P.I.) variations in pure TiO_2_ and composite forms with Zr, Zn, Ta, Fe, Cu, and Sn for tailored applications.

The Plasticity Index (P.I.) is a measure used in geotechnical engineering to assess the plasticity and workability of soils. It is calculated as the difference between the liquid limit (LL) and plastic limit (PL). A lower P.I. value generally indicates soils with less plasticity and better workability. In the context of the presented data in Fig. 13, where P.I. values are provided for different materials, a P.I. less than 0.8 is considered indicative of accurate preparations, particularly in soil stabilization and geotechnical applications^103^. When the P.I. is less than 0.8, it suggests that the soil or material has low plasticity, making it more stable and easier to work with. This is desirable in scenarios where soil stabilization is crucial, such as construction projects, where the soil needs to maintain its structural integrity^104^. Lower plasticity implies that the material is less prone to significant volume changes and is more resistant to deformation under external loads. In the context of the presented data, the materials with P.I. values less than 0.8, such as ZnO and Fe_2_O_3_, exhibit characteristics of accurate preparations for soil-related applications^105^. These materials could be considered for use in scenarios where low plasticity and improved workability are desired. It’s important to note that the interpretation of P.I. values may vary based on specific engineering requirements and the intended application of the materials. The variance (P.I.) variations depicted in Fig. 13 provide valuable insights into the tailored applications of pure TiO_2_ and its composite forms with Zr, Zn, Ta, Fe, Cu, and Sn. The Plasticity Index (P.I.) is a crucial parameter in geotechnical engineering, indicating the plasticity and workability of soils. Pure TiO_2_, represented by UV-100, exhibits a P.I. of 0.306, serving as a reference for comparison. Introducing different elements at a 0.1% concentration leads to varied changes in the Plasticity Index^106^. Notably, ZnO shows a substantial decrease in P.I., indicating a reduction in soil plasticity. ZrO and TiO_2_-CuO exhibit moderate increases, suggesting potential enhancements in soil workability. Ta_2_O_3_ and Fe_2_O_3_ demonstrate marginal changes, while SnO stands out with a significant increase in P.I., implying a notable impact on soil plasticity. These variations in P.I. are essential for tailoring the materials to specific applications, particularly in soil stabilization and geotechnical engineering. The observed trends in P.I. variations provide valuable information for optimizing compositions based on targeted functionalities, contributing to advancements in tailored applications for soil improvement and related fields^107^.

#### Av. Zeta potential measurements

**Fig. 14:**
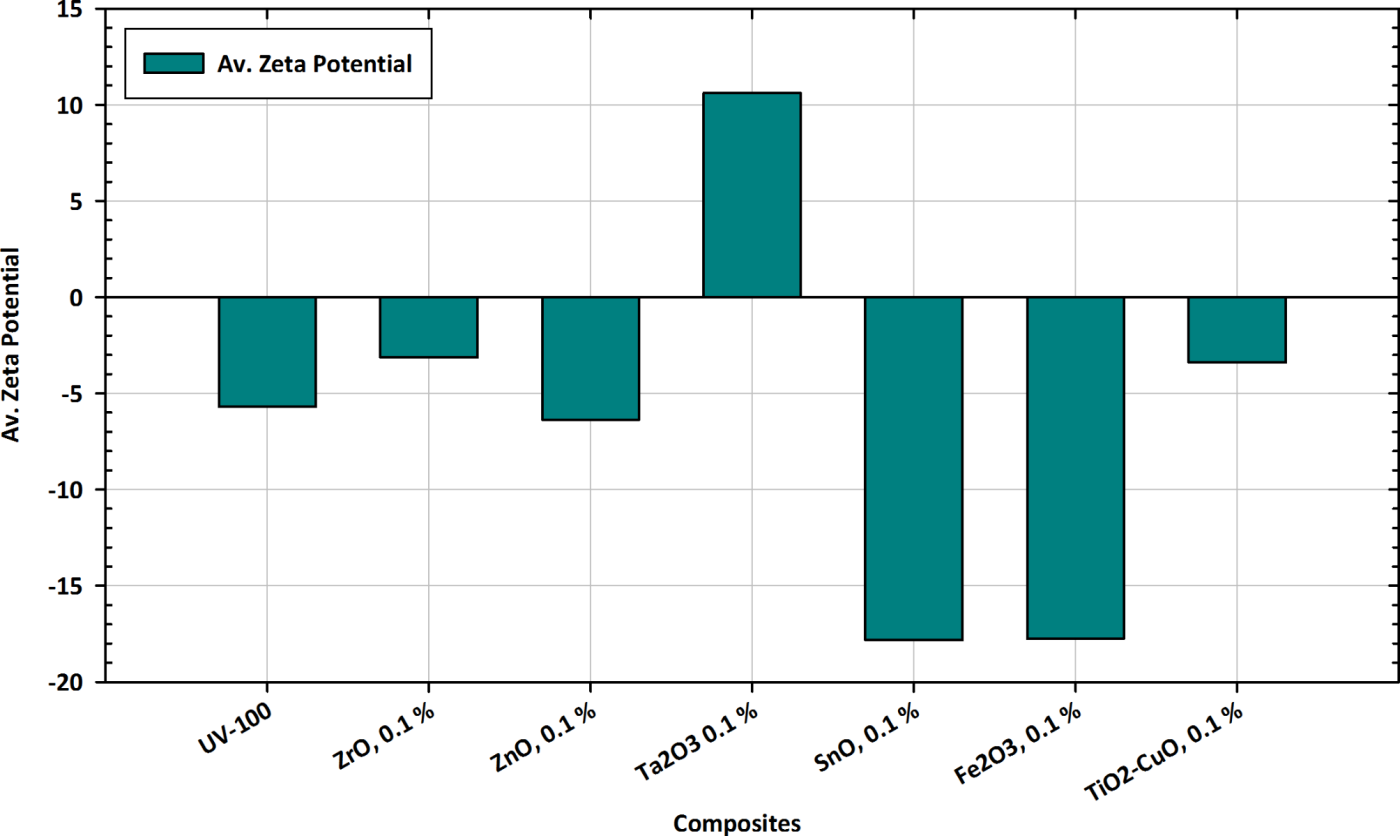
Av. Zeta Potential variations in pure TiO_2_ and composite forms with Zr, Zn, Ta, Fe, Cu, and Sn for tailored applications.

Zeta potential is an important parameter that indicates the surface charge of particles and their stability in a colloidal system. A higher absolute value of zeta potential typically suggests greater electrostatic repulsion between particles, leading to increased stability. In the context of the provided data in Fig. 14, the zeta potential values for different materials are presented. In the case of UV-100 (-5.7), the negative zeta potential indicates a moderate degree of stability. While not highly stable, the material still exhibits some electrostatic repulsion between particles^108^. For ZrO, 0.1% (-3.13), the negative zeta potential suggests moderate stability, like UV-100. However, the absolute value is slightly lower, indicating potentially reduced electrostatic repulsion. But in ZnO, 0.1% (-6.36), this material shows a more negative zeta potential, indicating higher stability than both UV-100 and ZrO. The increased absolute value suggests stronger electrostatic repulsion. In the case of Ta_2_O_3_ 0.1% (10.61), the positive zeta potential indicates instability, which might be attributed to a lack of electrostatic repulsion between particles. This material may tend to agglomerate. For SnO, 0.1% (-17.82), the highly negative zeta potential suggests excellent stability^109^. The material exhibits strong electrostatic repulsion, indicating potential dispersibility and resistance to agglomeration. But in Fe_2_O_3_, 0.1% (-17.74), like SnO, the highly negative zeta potential implies significant stability. The material is expected to resist agglomeration due to strong electrostatic repulsion. In the case of TiO_2_-CuO, 0.1% (-3.39), this material shows a negative zeta potential, indicating moderate stability like UV-100 and ZrO. In summary, materials with higher absolute zeta potential values (either positive or negative) generally exhibit greater stability, which is favorable for various applications where dispersion and prevention of agglomeration are essential. The specific application requirements and the desired behavior of the materials in each system will determine the suitability of each material based on its zeta potential^110^.

## Mechanistic insight

The modification of titanium dioxide (TiO_2_) with specific metal oxides has been widely studied to improve its photocatalytic performance by enhancing charge separation, altering the band structure, and extending the light absorption range. Firstly, the modification of the band structure starting with band gap narrowing since the metal oxides like ZnO, Fe_2_O_3_, or CuO can narrow the band gap of TiO_2_ by introducing new energy levels between the conduction band (CB) and valence band (VB) of TiO_2_. This allows for visible light absorption and makes the material more efficient under solar irradiation. For example, Fe_2_O_3_ has a smaller band gap (~ 2.1 eV) than TiO_2_ (~ 3.2 eV for anatase). When coupled with TiO_2_, Fe_2_O_3_ can introduce mid-gap states, shifting TiO_2_’s band gap towards the visible region, making it more photoactive under visible light. Secondly, the band alignment for charge transfer is clearly noticed when specific metal oxides form heterojunctions with TiO_2_, their conduction and valence bands align in such a way that electrons and holes are preferentially transferred between the two materials. In a typical type-II heterojunction, the conduction band of TiO_2_ is higher than that of the metal oxide (e.g., ZnO/TiO_2_). This allows electrons excited in TiO_2_ to transfer to the metal oxide, facilitating charge separation by preventing recombination. For Z-scheme heterojunction the configuration (e.g., Fe_2_O_3_/TiO_2_), the electrons in the conduction band of TiO_2_ reduce the recombination of holes from the valence band of the metal oxide, and the holes from TiO_2_ recombine with electrons from the metal oxide. This helps preserve high redox potential. Othe important issue is facilitating charge separation through electron sink effect since the metal oxides with lower Fermi levels, such as WO_3_, ZnO, or SnO_2_, act as electron acceptors, capturing photo-generated electrons from TiO_2_. This process effectively separates the electron-hole pairs and reduces the recombination rate. For example, WO_3_/TiO_2_ heterojunction, the WO_3_ has a conduction band potential more positive than TiO_2_, allowing it to act as an electron sink. When coupled with TiO_2_, WO_3_ captures electrons, leaving holes in the TiO_2_ to participate in oxidation reactions. We also facilitating charge separation through schottky barrier formation, since some metal oxides (e.g., ZnO, SnO_2_) form Schottky junctions with TiO_2_, which facilitate charge separation by creating an energy barrier that impedes the flow of electrons back into TiO_2_, preventing recombination. For example, the ZnO/TiO_2_ heterostructures often form Schottky-like junctions that efficiently separate charges due to a mismatch in their Fermi levels, with ZnO acting as an electron acceptor. One of the most important mechanisms is interfacial charge transfer mechanisms by firstly direct electron transfer since metal oxides can enable direct charge transfer at the interface between the two materials. Electrons excited in TiO_2_ can directly transfer to the metal oxide, where they participate in reduction reactions, while holes remain in TiO_2_ for oxidation processes. For example, in CuO/TiO_2_ composites, CuO acts as a p-type semiconductor with a conduction band below that of TiO_2_. Electrons excited in TiO_2_ can be transferred directly to CuO, enhancing charge separation. Secondly, through photoinduced interfacial charge transfer (PICT), since some metal oxides (like Co_3_O_4_ or Fe_2_O_3_) can absorb visible light and transfer the photoexcited electrons to the conduction band of TiO_2_, while holes remain in the metal oxide. This enhances the charge separation due to the movement of electrons to TiO_2_ and the subsequent delay in electron-hole recombination. Other proper mechanisms could be detected by enhancing visible light absorption through metal oxide sensitization since the narrow-band-gap metal oxides (e.g., Fe_2_O_3_, CuO, BiVO₄) are coupled with TiO_2_, they act as sensitizers. These oxides absorb visible light and inject electrons into the conduction band of TiO_2_, thereby utilizing a broader portion of the solar spectrum. For example, the CuO has a band gap of ~ 1.2 eV and can absorb visible light. When coupled with TiO_2_, CuO transfers its excited electrons to TiO_2_, which extends the photocatalytic response of TiO_2_ into the visible light region. For plasmonic effects, some doped metal oxides (e.g., Au-doped TiO_2_) can exhibit plasmonic effects, whereby localized surface plasmon resonance (LSPR) enhances visible light absorption and creates strong electric fields at the interface of the metal oxide and TiO_2_. This enhances charge separation and promotes photocatalysis. For illustration of increasing oxygen vacancies and defect sites it is very important to make creation of oxygen vacancies by doping or coupling TiO_2_ with certain metal oxides (e.g., CeO_2_, ZnO) introduces oxygen vacancies or other defects. These defects act as electron traps, which can prolong the lifetime of charge carriers by separating the electron-hole pairs, thus enhancing the photocatalytic efficiency. For example, the CeO_2_ can undergo redox cycles between Ce³⁺ and Ce⁴⁺, creating oxygen vacancies. When CeO_2_ is coupled with TiO_2_, the oxygen vacancies in CeO_2_ can trap photo-excited electrons, reducing electron-hole recombination and promoting enhanced photocatalytic activity. For reduction and recombination through multi-electron transfer pathways, the metal oxides (e.g., Co_3_O_4_, MnO_2_), electrons can follow multiple pathways, which increase the chances of charge separation and reduce the recombination rate. Such as the Co_3_O_4_ can facilitate multi-electron transfer due to its mixed-valent structure (Co^2+^/Co^3+^), allowing for enhanced electron flow from TiO_2_ to the metal oxide, which reduces recombination and improves photocatalytic efficiency. These mechanistic insights highlight how specific metal oxides significantly influence the performance of TiO_2_ in energy and environmental applications by enhancing charge separation and modifying the band structure for more effective photocatalysis.

## Suggested future work

Future studies can focus on optimizing the morphology of CuO-TiO_2_ composites (e.g., nanotubes, nanowires, or 2D sheets) to enhance surface area and improve photocatalytic efficiency. Advanced synthesis techniques like hydrothermal methods or sol-gel processing could be employed. Doping CuO-TiO_2_ with other metal oxides (e.g., ZnO, WO_3_) or non-metals (e.g., nitrogen or carbon) could further tune the band structure and enhance charge separation. Surface modifications with organic linkers or functional groups could also improve photocatalytic properties and stability. Developing multi-layer heterojunctions incorporating CuO-TiO_2_ with other semiconductors like MoS_2_ or g-C_3_N₄ could further enhance charge separation and extend visible light absorption. Investigating the interaction at the CuO-TiO_2_ interface could lead to more efficient electron-hole separation mechanisms. Future work could explore optimizing CuO-TiO_2_ for large-scale hydrogen production from water splitting. This includes testing under real-world conditions (solar illumination, continuous flow systems) and investigating stability over long periods. Investigating CuO-TiO_2_ composites in photoelectrochemical (PEC) cells for water splitting or photovoltaic cells could lead to more efficient solar-to-hydrogen conversion systems. Additionally, these materials could be used in dye-sensitized solar cells (DSSCs) or perovskite solar cells to improve efficiency. Further research could explore CuO-TiO_2_ as a corrosion inhibitor in aggressive environments like saline or acidic solutions. Studies should focus on applying materials as coatings for metals like steel, aluminum, and copper to investigate long-term performance, durability, and real-world compatibility in industries such as oil and gas, marine, and construction. Testing CuO-TiO_2_ composites for environmental remediation (e.g., degradation of organic pollutants, heavy metal removal) in real-world conditions (e.g., varying pH, temperature, or pollutant concentrations) could provide insights into scalability. Future studies could also investigate combining CuO-TiO_2_ with adsorption materials for enhanced removal efficiency of contaminants. Future work should focus on scalable, cost-effective synthesis methods for producing CuO-TiO_2_ composites in large quantities. Techniques like continuous flow reactors or industrial-scale sol-gel methods should be optimized for industrial production. For applications involving environmental and water purification, it is important to investigate the toxicity and environmental safety of CuO-TiO_2_ composites. Future work could involve detailed toxicity studies on the long-term release of Cu^2+^ ions and their effects on ecosystems and human health.

### Challenges and limitations

CuO-TiO_2_ composites demonstrate significant potential in various applications, several challenges and limitations remain, particularly regarding their scalability and practical deployment in real-world settings. Laboratory-scale synthesis methods such as hydrothermal processes or sol-gel techniques can produce CuO-TiO_2_ composites with high efficiency, these methods often involve expensive precursors, long reaction times, and high energy inputs. Scaling up to industrial-scale production while maintaining the material’s performance and quality can be cost-prohibitive. Ensuring the uniformity and consistency of CuO-TiO_2_ nanostructures across large production batches is a significant challenge. Variations in particle size, morphology, and composition could lead to inconsistent photocatalytic or electrochemical performance in real-world applications. One of the common challenges for TiO_2_-based photocatalysts, including CuO-TiO_2_, is photocorrosion, where the material degrades under prolonged exposure to light. This reduces the long-term stability of the material, particularly in applications like water splitting or environmental remediation, where long-term operation is required. CuO-TiO_2_ composites may suffer from reduced performance in real-world conditions, such as fluctuating temperatures, humidity, and exposure to different types of pollutants or ions. Ensuring long-term stability in harsh environments, like industrial effluent treatment plants or marine conditions, remains a challenge. Despite CuO’s ability to improve charge separation in TiO_2_, recombination of electron-hole pairs remains an issue, particularly under real-world sunlight exposure. Improving electron transfer efficiency and further reducing recombination is critical to improving photocatalytic and energy conversion efficiency. CuO can extend the light absorption range of TiO_2_ into the visible region, the overall efficiency of visible light utilization is still limited. Finding the right balance between CuO loading and TiO_2_ structure to optimize the band gap while maintaining stability and avoiding excess recombination is a challenge. CuO-TiO_2_ composites show great potential in lab settings, integrating them into existing industrial systems (e.g., water treatment plants, solar cells, or anti-corrosion coatings) may require significant infrastructure modification, which could be costly and time-consuming. For instance, adapting CuO-TiO_2_ materials to work efficiently in large-scale photoelectrochemical cells or reactors could face technical and engineering hurdles. The scalability of CuO-TiO_2_ composites is also hindered by the costs associated with copper oxides and high-purity titanium precursors. Reducing the cost of these materials while maintaining performance is essential for large-scale adoption. One of the concerns with CuO-TiO_2_ composites, especially in environmental applications, is the potential release of Cu^2+^ ions into the environment, which could have toxic effects on aquatic organisms or ecosystems. This is especially important for applications like water purification or corrosion prevention in marine environments, where Cu^2+^ release needs to be tightly controlled. The potential toxicity of nanomaterials, including CuO-TiO_2_, to both humans and the environment remains an underexplored area. Before large-scale application, there was a need for in-depth environmental and health risk assessments to ensure that the widespread use of these composites does not lead to unintended negative consequences. In environmental remediation applications, CuO-TiO_2_ composites may degrade over time due to fouling, clogging, or accumulation of pollutants on the surface, leading to a decrease in efficiency. Ensuring the reusability of these materials without significant performance loss is an important challenge to address. In water treatment or photocatalytic degradation systems, recovering CuO-TiO_2_ nanoparticles after use can be challenging, especially if the particles are suspended in water. Efficient methods for recovering or regenerating the material after use are needed to minimize environmental impact and operational costs. CuO-TiO_2_ composites have demonstrated promising results in lab-scale hydrogen production from water splitting, their efficiency under natural sunlight is still below the level required for large-scale energy production. The materials may also suffer from degradation under continuous operation, reducing their practical viability for long-term hydrogen generation. Most studies on CuO-TiO_2_ composites have focused on short-term performance. The long-term behavior of these materials under real-world conditions (e.g., continuous solar exposure, high pollutant loads, or varying pH and temperature) is still not well understood. Long-term studies are needed to evaluate the durability, performance, and potential degradation pathways of these composites over time. Different applications (e.g., photocatalysis, energy storage, anti-corrosion) may require different design optimizations for CuO-TiO_2_ composites. Tailoring the band structure, surface chemistry, and morphology for each specific use case remains a challenge. For instance, optimizing photocatalytic efficiency may not align with the requirements for corrosion inhibition, making it difficult to develop a one-size-fits-all material.

## Conclusion

The comparative study conducted on various photocatalytic materials, including TiO_2_/ZrO_2_, ZnO, Ta_2_O_3_, SnO, Fe_2_O_3_, and CuO-based composites, provides valuable insights into their enhanced photocatalytic applications. The investigation encompassed a comprehensive analysis of multiple properties, including morphological insights, surface area variations, pore size, particle size, Variance (P.I.), and Zeta potential. The findings contribute to our understanding of the potential of these materials for tailored applications. The transmission electron microscopy (TEM) images revealed the morphological characteristics of the materials. The variations in morphology can influence the photocatalytic performance, with different materials showing distinct features. The surface area is a crucial factor influencing photocatalytic activity. Materials with larger surface areas, such as ZnO, may exhibit enhanced photocatalytic performance due to increased active sites for reactions. Pore size is another determinant of the materials’ photocatalytic efficiency. Materials with optimal pore sizes, such as SnO, could facilitate better diffusion of reactants to active sites, enhancing the overall performance. Particle size influences the materials’ reactivity. Smaller particle sizes, as observed in Fe_2_O_3_, may result in higher surface areas, and increased photocatalytic activity. The low values of Variance (P.I.) for ZnO and Fe_2_O_3_ indicate accurate preparations, suggesting their potential reliability and reproducibility in applications. Zeta potential values indicate the stability of colloidal particles. Materials with higher absolute zeta potential values, such as SnO and Fe_2_O_3_, exhibit greater stability, which is beneficial for applications requiring dispersion. Finally, comparative study allows for a nuanced understanding of the materials’ characteristics and their implications for photocatalytic applications. Depending on specific application requirements, such as pollutant degradation or water purification, researchers and engineers can make informed choices based on the observed variations in properties. The study opens avenues for further exploration and optimization of these materials for tailored photocatalytic processes, contributing to advancements in environmental and energy-related applications. Results revealed that the photo-activity of all prepared composites were more effective than the photo –activity of commercial hombikat UV-100. The rate of efficiency is arranged in the order TiO_2_/CuO > TiO_2_/SnO > TiO_2_/ZnO > TiO_2_/Ta_2_O_3_ > TiO_2_/ZrO_2_ > TiO_2_/Fe_2_O_3_ > Hombikat TiO_2_-UV100 the photodegradation and removal of Imazapyr were achieved as 79.5%, 77.5%, 75.4%, 66.3%, 63%, 51.2% and 50.6% respectively. From these results, all composites exhibited superior performance, attributed to enhanced light absorption and charge separation.

## Data Availability

All data are included in the manuscript.
